# *CACNA1C* gene regulates behavioral strategies in operant rule learning

**DOI:** 10.1371/journal.pbio.2000936

**Published:** 2017-06-12

**Authors:** Georgia Koppe, Anne Stephanie Mallien, Stefan Berger, Dusan Bartsch, Peter Gass, Barbara Vollmayr, Daniel Durstewitz

**Affiliations:** 1 Department of Theoretical Neuroscience, Bernstein Center for Computational Neuroscience, Central Institute of Mental Health, Medical Faculty Mannheim of Heidelberg University, Mannheim, Germany; 2 Clinic for Psychiatry and Psychotherapy, Central Institute of Mental Health, Medical Faculty Mannheim of Heidelberg University, Mannheim, Germany; 3 Department of Molecular Biology, Central Institute of Mental Health, Medical Faculty Mannheim of Heidelberg University, Mannheim, Germany; Institute of Science and Technology Austria, Austria

## Abstract

Behavioral experiments are usually designed to tap into a specific cognitive function, but animals may solve a given task through a variety of different and individual behavioral strategies, some of them not foreseen by the experimenter. Animal learning may therefore be seen more as the process of selecting among, and adapting, potential behavioral policies, rather than mere strengthening of associative links. Calcium influx through high-voltage-gated Ca^2+^ channels is central to synaptic plasticity, and altered expression of Ca_v_1.2 channels and the *CACNA1C* gene have been associated with severe learning deficits and psychiatric disorders. Given this, we were interested in how specifically a selective functional ablation of the *Cacna1c* gene would modulate the learning process. Using a detailed, individual-level analysis of learning on an operant cue discrimination task in terms of behavioral strategies, combined with Bayesian selection among computational models estimated from the empirical data, we show that a *Cacna1c* knockout does not impair learning in general but has a much more specific effect: the majority of *Cacna1c* knockout mice still managed to increase reward feedback across trials but did so by adapting an outcome-based strategy, while the majority of matched controls adopted the experimentally intended cue-association rule. Our results thus point to a quite specific role of a single gene in learning and highlight that much more mechanistic insight could be gained by examining response patterns in terms of a larger repertoire of potential behavioral strategies. The results may also have clinical implications for treating psychiatric disorders.

## Author summary

To deal with an uncertain and complex world, animals have developed a large repertoire of behavioral heuristics and default strategies that spring into action in unknown situations. Building on this a priori repertoire, animals may find various ways to succeed on a given behavioral task. Therefore, determining the exact behavioral strategy followed during a task may be essential for understanding the cognitive processes involved. Using computational models to analyze behavior, we examined how a genetic variation in a gene that encodes a calcium channel and has been associated with learning deficits influences the way in which animals acted on a task in which a reward was associated with a specific behavior. We found that a knockout of the relevant gene does not lead to a general learning impairment but rather led animals to adopt a behavioral strategy different from the one employed by the control animals. Specifically, knockout animals managed to increase their reward returns by basing their responses more on the previous reward location rather than on reward-indicating stimuli, like the controls did. These findings may prove useful for behavioral therapy in the context of psychiatric disorders associated with this specific gene variation.

## Introduction

The ability to learn, to adapt one’s own behavior in order to optimize positive and avoid negative feedback, is central to all living beings. Animals like humans are constantly seeking to infer the (causal) structure of their environment and to predict the outcomes of their actions [1,2], sometimes to a degree that “superstitial beliefs” about environmental contingencies may form [3]. However, since the environment is only partially observable, with an often infinite or exponentially exploding space of possibilities, inferring the optimal course of actions is, in general, a computationally intractable problem [4]. Hence, animals like humans rely on heuristics, strategies, and ecological biases that favor certain types of environmental contingencies over others and narrow down the search space [5,6]. For instance, rodents are predisposed to employ ecologically sensible strategies like win-stay, lose-shift, or alternate [7–9], and these behaviors are also more readily acquired [10,11]. Moreover, recent evidence suggests that animal learning, even on apparently simple conditioning tasks, may often engage active, evidence-driven choices among behavioral strategies, rather than mere passive strengthening of stimulus-response (SR) associations [12,13]. The upshot here is that animal learning may rely on a large variety of different previously acquired or predisposed strategies, rather than on a uniform mechanism, like SR strengthening, that could either be enabled or disabled.

Given this larger repertoire of potential a priori strategies and heuristics with which animals may enter any given experimental task context, there may be more than one way for increasing reward [14,15], even though this may not have been experimentally intended. In such situations, more conventional analysis in terms of error counts or reaction times compared among experimental groups may perhaps (wrongly) infer that one group is simply diminished in its learning abilities compared to another, while the observed differences may actually be rooted in the different behavioral strategies applied. Individual differences in learning strategies have recently been approached within the framework of computational reinforcement learning (RL) and decision-making theories [e.g., 16,17], which have been applied, for instance, to disentangle model-free and model-based forms of learning and their distinct neural substrates [18–20] or to reveal strategic interactions during (model-based) sequential planning [21]. Building on ideas like these, we show here how a genetic variation may determine an animal’s learning strategy and associated performance patterns. Specifically, we studied learning in terms of behavioral strategies on a 2-choice operant cue discrimination task in 2 groups of mice, a group with a genetic modification associated with severe learning deficits [22], namely, a selective knockout (KO) of the *Cacna1c* gene coding for the α subunit of Ca_v_1.2 L-type high-voltage-dependent calcium channels (Ca_v_1.2^NesCre^), and matched controls with intact gene expression (Ca_v_1.2^fl/fl^). Calcium influx through voltage-dependent calcium channels is essential for synaptic plasticity and other cellular adaptations [23] thought to underlie instrumental learning [24]. The *CACNA1C* gene has also been implicated in the pathophysiology of psychiatric disease [22,25], rendering it an important target for understanding learning mechanisms not only in normal but also psychiatric conditions. By studying the detailed pattern of behavioral responses and rewards received across the learning process, combined with analyses based on behavioral (reinforcement) learning models, we found that Ca_v_1.2^NesCre^ mice did not simply exhibit a general learning deficit but rather relied on different behavioral strategies than Ca_v_1.2^fl/fl^ mice.

## Results

### Ca_v_1.2^NesCre^ and Ca_v_1.2^fl/fl^ mice both improve performance, but to differing degrees and with different patterns

**Fig 1:**
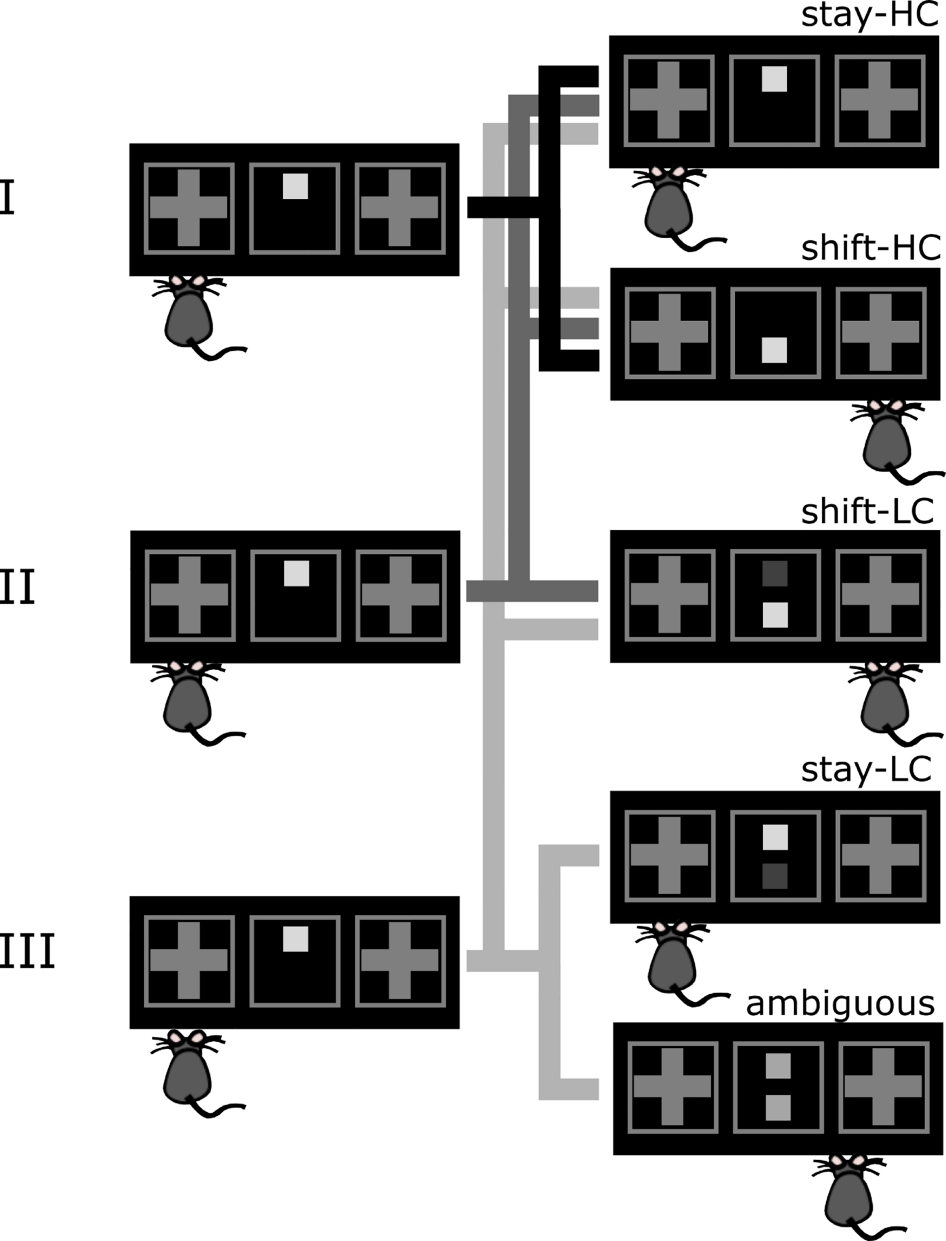
Two-choice operant cue learning task. A square-shaped cue appears in either the top or bottom position of a central field, indicating a correct left/right side response. A “stay trial,” as defined here, requires a response to the same side as on the previous trial, while a “shift trial” requires a response to the opposite side (given a correct previous response). The figure displays the different trial types added in successive task phases (labeled I, II, and III). In phase I, only high-contrast (HC) cues were presented. In phases II and III, low-contrast (LC) cues were added. Phase III further included completely ambiguous trials for which both response options were rewarded.

To examine potential learning deficits caused by selective ablation of the Ca_v_1.2 L-type calcium channel subunit, a cue discrimination task consisting of 3 different task phases was set up (Fig 1). The basic task required animals to perform a nose poke response on one side of a touchscreen box if a light square came up in the upper central position of the screen and to the other side if the stimulus was shown in the lower position (Fig 1I). Hence, the animals had to associate cue location (top, bottom) with response side (left, right) for a maximum of reward returns (correct responses according to this “cue rule” were always rewarded). In task phase I, trials with the cue in top or bottom position occurred in a pseudorandom order (see Materials and methods for details), but correction trials consisting of repetitions of the unsuccessful trial were run after each wrong (nonrewarded) response. In task phase II, “slightly ambiguous” trials with reduced stimulus contrast were introduced, i.e., with cues appearing in both positions but the one indicating the correct response side brighter than the other (Fig 1II). In phase III, “fully ambiguous” trials were added with cues in both positions with the same brightness (responses to both sides were rewarded in this case [Fig 1III]). Also, in phases II and III, trials which required animals to perform responses to the side opposite to the one rewarded in the previous trial (termed “shift trials” here) were always followed by 1–2 trials in which the cue indicated the side rewarded in the previous trial (“stay trials”) (see Materials and methods and S1 Fig for details). As detailed below, these manipulations in phases II and III served as additional probes for the behavioral strategies or biases exhibited by the animals (except for the analyses targeting specifically these trials, however, different trial types were pooled for analysis whenever possible). They were introduced in sequential task stages and not all at once so as to not overload the early learning phase for the animals. No significant side bias was found in either group of animals on the first day of testing, nor was there a difference in side preference between groups (see Materials and methods).

**Fig 2:**
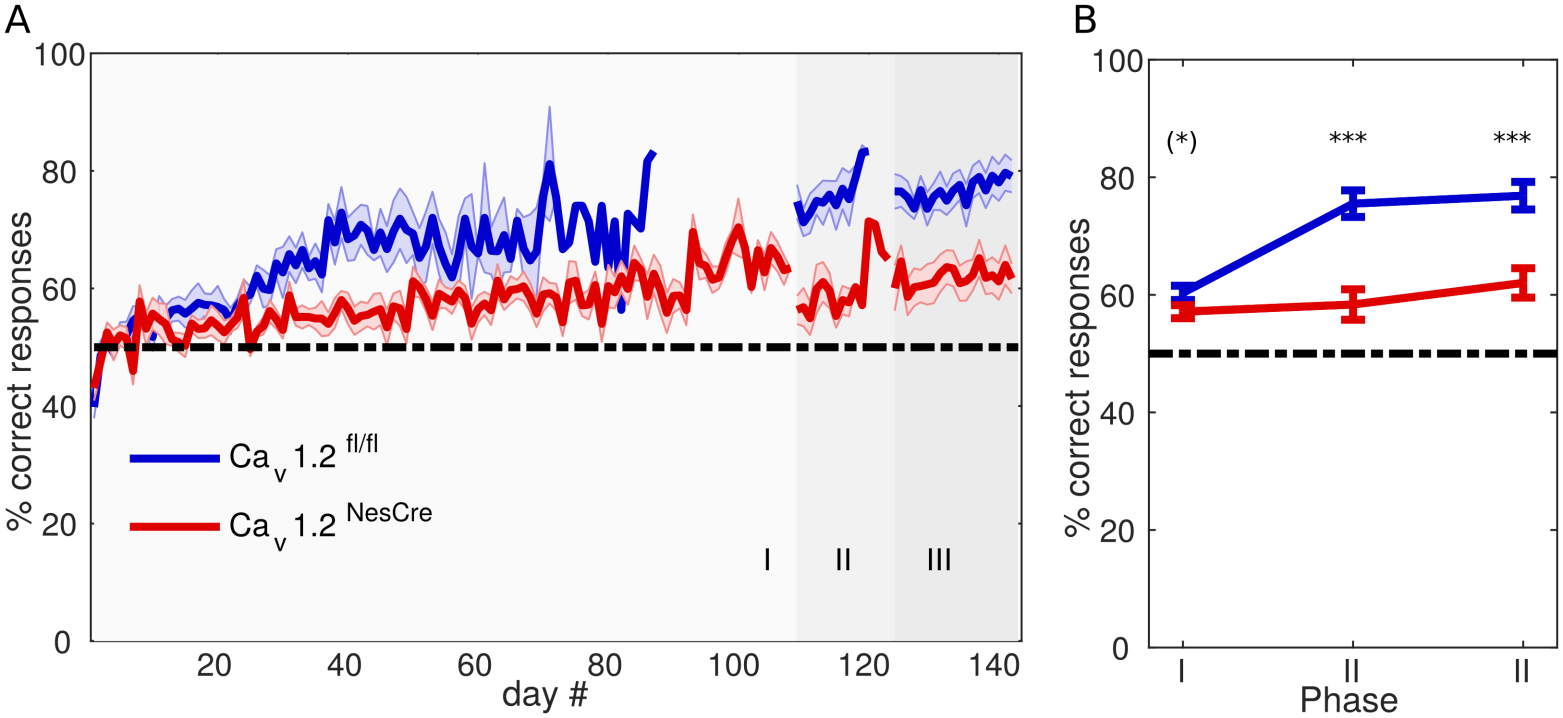
Cue-rule-consistent (i.e., correct) responses across task phases and days. A) Mean and SEM of the percentage of correct responses by day for control (Ca_v_1.2^fl/fl^; blue) and Ca_v_1.2 knockout (Ca_v_1.2^NesCre^; red) mice. Different gray shadings indicate the 3 task phases. Note that Ca_v_1.2^fl/fl^ mice reached the experimenter-defined performance criterion earlier than Ca_v_1.2^NesCre^ mice in phase I and therefore were recorded for fewer days. Binomial tests revealed significant improvements above chance in 11/12 Ca_v_1.2^NesCre^ and 12/12 Ca_v_1.2^fl/fl^ animals in phase I, 7/11 Ca_v_1.2^NesCre^ and 11/12 Ca_v_1.2^fl/fl^ in phase II, and 11/11 Ca_v_1.2^NesCre^ and 12/12 Ca_v_1.2^fl/fl^ in phase III. This was further supported by group-level comparisons by *t* tests on the last day of each task phase against chance: all *p <* .01, and last day against first day of phase I: p < .001 for both groups. B) Percentages of correct responses averaged across each task phase (error bars = SEM). Asterisks mark significant differences between groups as revealed by post hoc tests: Ca_v_1.2^fl/fl^ performed significantly better than Ca_v_1.2^NesCre^ mice in phases II and III (*p <* .001) but only marginally better in phase I (*p* = .088). Data available at https://github.com/GKoppe/BehavioralData_Ana.

First, we assessed the number of correct responses according to the experimenter-defined “cue rule” (Fig 2). Both groups of animals, Ca_v_1.2^NesCre^ and Ca_v_1.2^fl/fl^, displayed levels of accuracy as defined by this rule that ranged significantly above chance in all 3 task phases, as confirmed by both subject-level binomial and group-level *t* tests (see Fig 2 legend for statistical details). This verifies that performance levels in both groups significantly increased over the course of the task, even if assessed purely in terms of the experimenter-defined cue rule. Nevertheless, a 2 × 3-factorial analysis of variance (ANOVA) with “group” (Ca_v_1.2^NesCre^ versus Ca_v_1.2^fl/fl^) as between- and “task phase” as within-subjects factor revealed a main effect of group (*F*(1,21) = 20.19, *p <* .001), a main effect of task phase (*F*(2,42) = 40.62, *p <* .001), and an interaction effect (*F*(2,42) = 18.7, *p <* .001), indicating that performance was significantly worse for Ca_v_1.2^NesCre^ compared to Ca_v_1.2^fl/fl^ animals, in a task-phase-dependent manner, as further confirmed by post hoc tests (Fig 2B; note that for these across-phase comparisons low- and high-contrast trials were combined in phases II & III, as these were not present in phase I, see Materials and methods and below).

**Fig 3:**
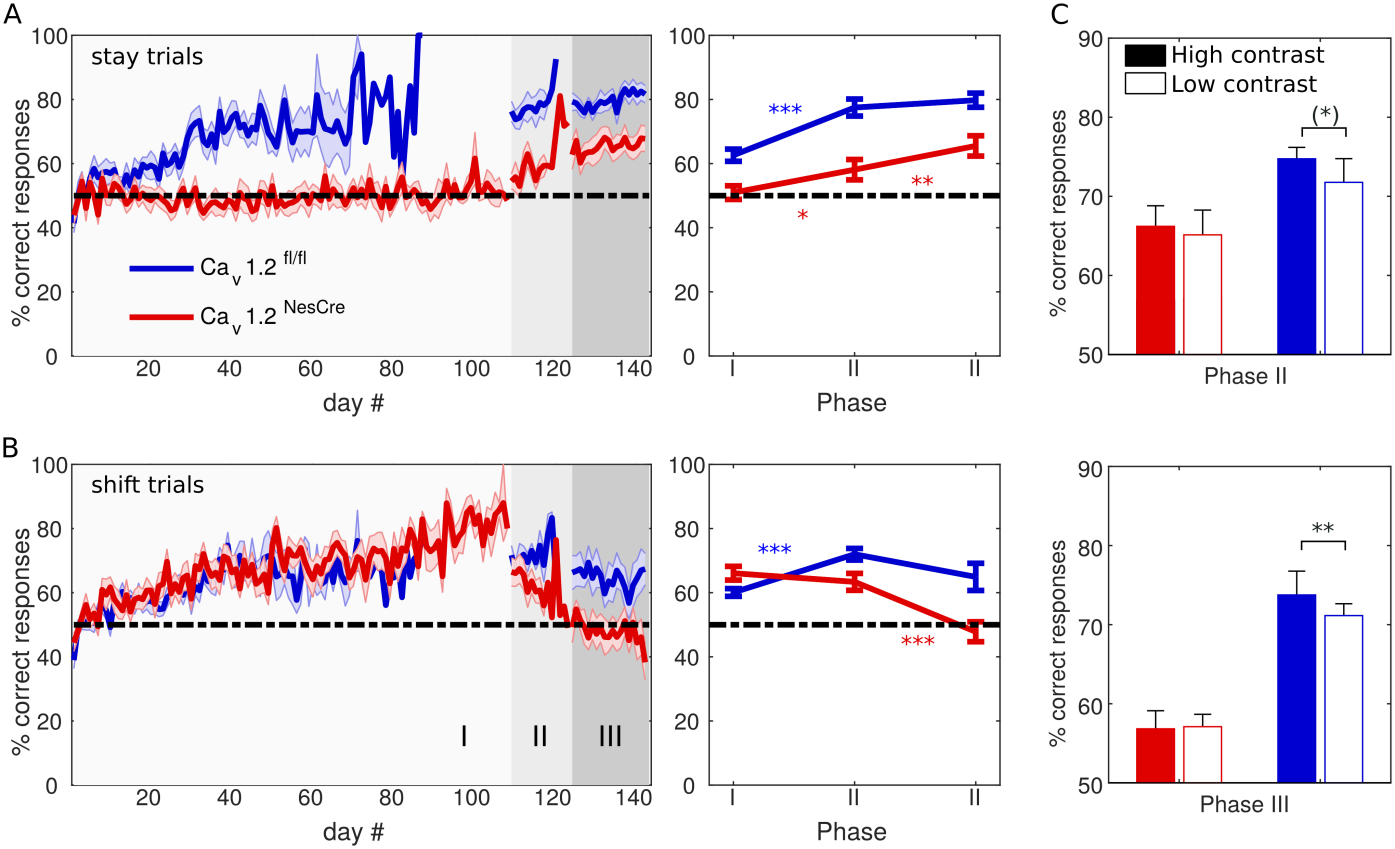
Cue-consistent responses across task phases and days split for stay and shift trials as defined in Fig 1. Mean and SEM of percentage of correct responses per day (left) and averaged across task phases (right) for Ca_v_1.2^fl/fl^ (blue) and Ca_v_1.2^NesCre^ (red) mice on A) stay trials and B) shift trials. Different gray shadings indicate the three task phases. C) Percentage of correct responses on high contrast (solid bars) and low contrast (empty bars) in phase II (top) and III (bottom), color-coded as in A. Error bars = SEM. Data available at https://github.com/GKoppe/BehavioralData_Ana.

Next, we examined whether performance depended on whether the previous trial required a response to the same side for obtaining reward as the current one (“stay trial”) or a response to the opposite side (“shift trial”). For both shift and stay trials, we again observed a main effect of group (shift trials: *F*(1,21) = 6.55, *p* = .018, stay trials: *F*(1,21) = 22.59, *p <* .001), a main effect of task phase (shift trials: *F*(1.35,28.33) = 11.12, *p* = .001, stay trials: *F*(2,42) = 47.97, *p <* .001), and a significant phase-by-group interaction (shift trials: *F*(1.35,28.33) = 11.01, *p* = .001, stay trials: *F*(2,42) = 3.54, *p* = .038). While Ca_v_1.2^fl/fl^ mice essentially showed the same performance pattern across task phases in both types of trials (i.e., an increase from phase I to phase II, both post hoc tests *p <* .001, and no further increase in phase III, both *p >* .1), as they should if they acted according to the cue rule, for Ca_v_1.2^NesCre^, the response pattern strongly differed on shift and stay trials: while, in fact, Ca_v_1.2^NesCre^ animals performed around chance level in stay trials throughout the whole first task phase before jumping to higher performance levels in phases II and III (post hoc tests phase I versus II: *p* = .033, phase II versus III: *p* = .003, see Fig 3A), they demonstrated high performance on shift trials in phase I, which then, however, dramatically declined across task phases (post hoc tests phases II versus III: *p* = .001, see Fig 3B). Indeed, Ca_v_1.2^NesCre^ even outperformed Ca_v_1.2^fl/fl^ animals on shift trials during phase I (post hoc tests Ca_v_1.2^fl/fl^ versus Ca_v_1.2^NesCre^ phase I: *p* = .044), while Ca_v_1.2^fl/fl^ outperformed Ca_v_1.2^NesCre^ in all other conditions (all *p <* .05).

The fact that Ca_v_1.2^NesCre^ animals did not exceed chance level in terms of the cue rule throughout stay trials in phase I and shift trials in phase III suggests that they may not have gathered the experimentally intended cue-response association. This interpretation is further supported by a separate analysis of trials with reduced stimulus contrast (Fig 3C): while Ca_v_1.2^fl/fl^ animals were significantly influenced by this manipulation, as one would expect if their behavior were controlled by the cue (low- versus high-contrast trials, phase II: *t*(11) = 1.82, *p* = .096; phase III: *t*(11) = 3.34, *p* = .007), reducing cue contrast did not affect the behavior of the Ca_v_1.2^NesCre^ animals (low- versus high-contrast trials, phase II: *t*(10) = .33, *p* = .75; phase III: *t*(10) = –.21, *p* = .836).

### Ca_v_1.2^NesCre^ and Ca_v_1.2^fl/fl^ mice may follow different behavioral strategies for increasing reward rate

**Fig 4:**
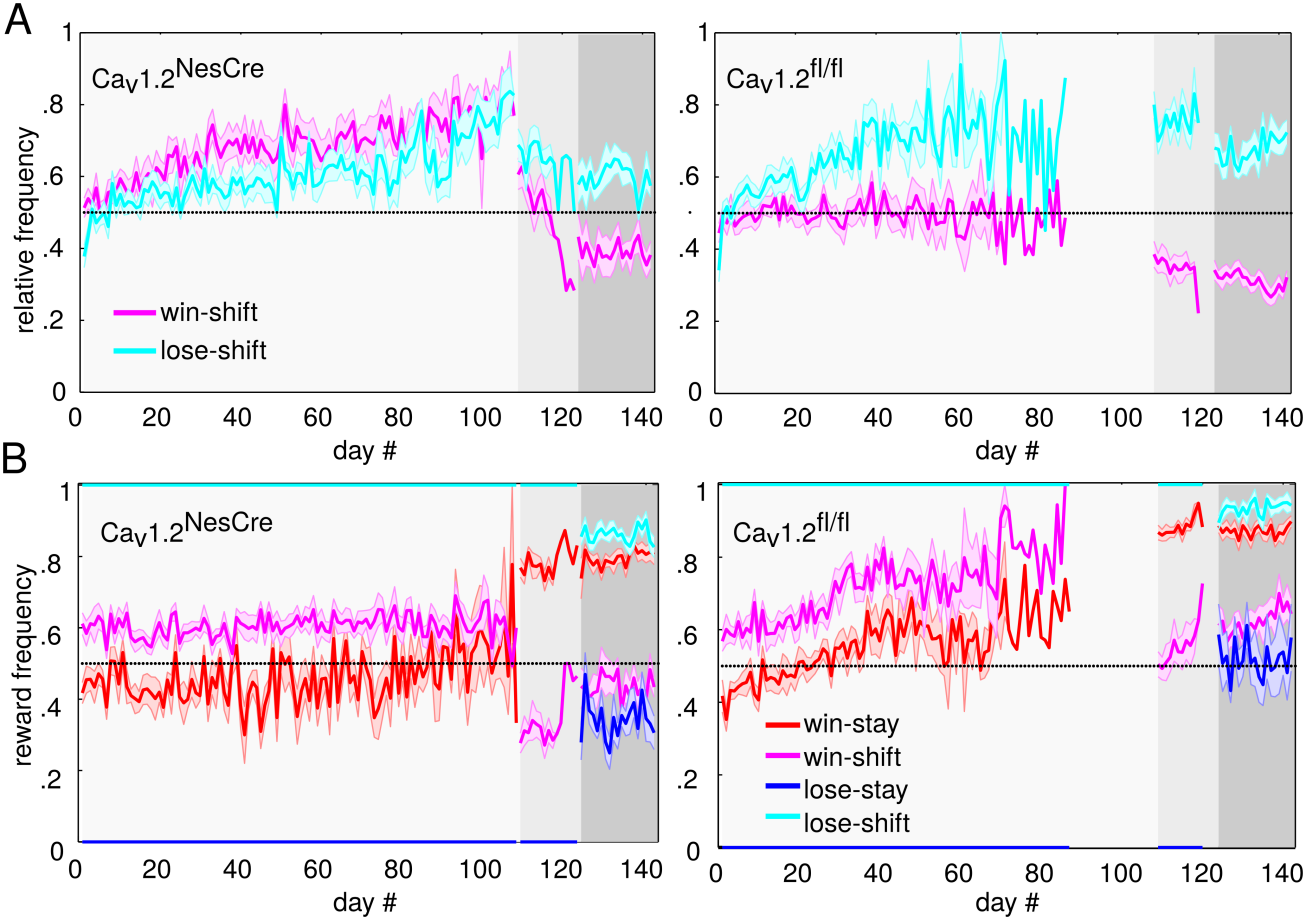
Task performance and reward feedback according to outcome-based rules. A) Relative frequency of win-shift (magenta) and lose-shift (cyan) responses per day for Ca_v_1.2^NesCre^ (left) and Ca_v_1.2^fl/fl^ (right) animals (error shadings = SEM). Different gray shadings indicate the three task phases. Note that win- and lose-stay frequencies are symmetric to win- and lose-shift frequencies about the 0.5-axis and are thus not shown. B) Relative frequency with which outcome-rule-consistent responses led to reward for Ca_v_1.2^NesCre^ (left) and Ca_v_1.2^fl/fl^ (right) animals. Data available at https://github.com/GKoppe/BehavioralData_Ana.

The results above raise the question of how the Ca_v_1.2^NesCre^ mice did manage to improve task performance, although they apparently did not issue their responses in accordance with the presented cue. In novel environments, rodents quickly adopt and learn to adapt ecologically prepared or previously acquired strategies like win-stay or win-shift [e.g. 7,10,11], which we will denote as “outcome rules” in the following, rather than extracting the (cue-based) rules experimentally imposed. To assess this, we first evaluated how consistent their pattern of responses is with any of the 4 elementary outcome-based strategies: win-stay, win-shift, lose-stay, and lose-shift. Fig 4A gives the relative frequency of responses across trials on each day that are consistent with a win-shift and a lose-shift rule for Ca_v_1.2^NesCre^ and Ca_v_1.2^fl/fl^ animals (note that by symmetry, *p*(win-shift) = 1–*p*(win-stay), and *p*(lose-shift) = 1–*p*(lose-stay)).

In general, there was a main effect of (outcome-based) strategy (*F*(1,21) = 78.03, *p <* .001) and task phase (*F*(1,21) = 78.03, *p <* .001), but the strategy depended on phase (strategy x phase: *F*(2,42) = 47.06, *p <* .001) and was further modulated by group (strategy x group: *F*(1,21) = 32.16, *p <* .001 and strategy x group x phase: *F*(2,42) = 3.306, *p* = .046): on the one hand, outcome-strategy-conforming responses appeared to change in both groups with task phase and trial history, but on the other, this adaptation was clearly different for Ca_v_1.2^NesCre^ and Ca_v_1.2^fl/fl^ animals. Specifically, both groups favored lose-shift over lose-stay, that is, tended to shift to the other side after an unsuccessful response (binomial tests on lose-shift against chance across all task phases: *p*≤.001 for 23/24 animals). After successful trials, however, Ca_v_1.2^NesCre^ mice considerably differed in their behavior from Ca_v_1.2^fl/fl^: During phase I, they shifted significantly more often after wins than Ca_v_1.2^fl/fl^ (post hoc test: *p* = .001) and even marginally more so than after losses (post hoc test win-shift versus lose-shift for Ca_v_1.2^NesCre^: *p* = .079), thus basically increasing their overall probability for shifting (Fig 4A left). Ca_v_1.2^fl/fl^ mice, in contrast, exhibited significantly more shifts after incorrect trials than after correct ones (post hoc test win-shift versus lose-shift for Ca_v_1.2^fl/fl^: *p <* .001), with no preference for win-shift over win-stay (see Fig 4A right, *p*(shift | win) ≈ .5, *t*(11) = –.02, *p* = .98). In phases II and III, Ca_v_1.2^NesCre^ animals then reduced shifting after correct but not incorrect responses (post hoc tests win-shift in phase I versus II: *p* = .012, phase II versus III: *p <* .001). Although this general trend is similar to that shown by Ca_v_1.2^fl/fl^ (phase I versus II and phase II versus III: *p <* .001), Ca_v_1.2^NesCre^ animals still win-shifted significantly more often than Ca_v_1.2^fl/fl^ in phase II (*p* = .001) and, by trend, in phase III (*p* = .069). Thus, in essence, Ca_v_1.2^NesCre^ animals increased the overall probability for shifting during phase I and then selectively and progressively decreased their win-shift tendency in the next 2 phases (see also S2 Fig).

Note that the larger proportion of stay-correct trials in task phases II and III by task design (see above and Materials and methods) makes downregulating win-shift responses a sensible strategy. This raises the question of whether the below-chance decrease in win-shift responses observed also in Ca_v_1.2^fl/fl^ animals reflects (partial) adoption of a win-stay strategy or whether this decrease could partly be related to other, confounding factors, like correlations among cue and outcome rules (overlap between cue and win-stay (outcome) rule: phase I: ~47%, phase II: ~74%, and phase III: ~82%). The ambiguous trials introduced in phase III (with equal cue intensities in the top and bottom positions) might help to dissociate cue- versus outcome-based rules, since, on these trials, the cue is completely noninformative with respect to response side. However, both Ca_v_1.2^NesCre^ and Ca_v_1.2^fl/fl^ animals kept on applying a win-stay strategy in these trials (Ca_v_1.2^NesCre^: 60% win-stay, *t*(10) = 2.80, *p* = .019 compared to chance; Ca_v_1.2^fl/fl^: 59%, *t*(11) = 2.84, *p* = .016), although Ca_v_1.2^fl/fl^ animals showed this behavior significantly less often than in other phase III trials (*t*(11) = –6.16, *p <* .001), while Ca_v_1.2^NesCre^ animals did not (*t*(10) = –1.39, *p* = .19). Ca_v_1.2^NesCre^ (but not Ca_v_1.2^fl/fl^) animals also exhibited a highly significant correlation between win-stay behavior on ambiguous and nonambiguous trials across days (Ca_v_1.2^NesCre^: *t*(10) = 6.59, *p <* .001, Ca_v_1.2^fl/fl^: *t*(11) = .82, *p* = .43, and Ca_v_1.2^NesCre^ versus Ca_v_1.2^fl/fl^: *t*(21) = 2.12, *p* = .046), further supporting the idea that Ca_v_1.2^NesCre^ (in contrast to Ca_v_1.2^fl/fl^) animals more generally followed an outcome rule. These observations thus suggest that, in truly ambiguous situations, Ca_v_1.2^fl/fl^ animals may also partly revert to a win-stay strategy. In situations like phase I, however, where win-staying bears no advantage over win-shifting, Ca_v_1.2^fl/fl^ (unlike Ca_v_1.2^NesCre^) animals did not show any such preference but followed the more rewarding cue rule (see above).

While decreasing win-shifting is sensible in phases II and III given the task design, it does not explain why Ca_v_1.2^NesCre^ animals clearly adapted their outcome-based behavior over time also in phase I or why, more generally, this might be a worthwhile approach in the present task. Ultimately, one would expect animals to increase the likelihood for a certain strategy if it turned out to be more rewarding than alternatives and, in particular, compared to random responding. We therefore next examined the frequency of rewards the Ca_v_1.2^NesCre^ animals had actually received on responses consistent with each of the 4 outcome-based response options (Fig 4B). Different outcome strategies were indeed associated with significantly different reward probabilities (main effect strategy: *F*(1,10) = 457.7, *p <* .001), and these were further modulated by task phase (strategy x task phase interaction: *F*(1.29,12.86) = 545.06, *p <* .001). While shifting after an incorrect trial was the most rewarding strategy (post hoc comparison of lose-shift to all others in all phases: *p <* .001), reward probability for shifting versus staying after a correct trial really depended on the experimental phase: a win-shift strategy was more rewarding in phase I (win-shift versus win-stay: *p <* .001) and less rewarding in phases II and III (post hoc tests both *p <* .001). Moreover, employing binomial tests, win-shift-consistent responses in phase I were significantly more often associated with reward than would have been expected by chance (for all Ca_v_1.2^NesCre^ animals: *p <* .001). Thus, based on the actual outcomes the animals had experienced, a win-shift/lose-shift strategy should have been perceived as more rewarding than either chance responding or any alternative combination of outcome-based responses in phase I, while win-stay/lose-shift would have been the most effective combination in phases II and III, consistent with the pattern of responses Ca_v_1.2^NesCre^ animals actually displayed (Fig 4A).

### Ca_v_1.2^NesCre^ behavior is best explained in terms of outcome rules and Ca_v_1.2^fl/fl^ behavior best in terms of the cue rule

The analyses in the previous sections showed that the actual response pattern in Ca_v_1.2^NesCre^ mice is more consistent with outcome-based rules, while that of Ca_v_1.2^fl/fl^ mice is more consistent with the cue rule, and that these strategies could have been perceived as rewarding by the animals based on the actual experiences they have had. To conclusively statistically demonstrate that outcome-based versus cue-based behavior indeed sufficiently explained the animal choices both across and within task phases, we next examined the animals’ behavior in terms of bootstrap distributions and formal reinforcement learning (RL) models generated from the empirical data.

An outcome rule bootstrap distribution was generated by having an “artificial agent” making choices purely based on the 4 outcome rules but with probabilities for the 4 response options (win-stay, win-shift, lose-stay, and lose-shift) derived from the animals’ actual choice frequencies on each day. Hence, this “agent” would choose to win-stay, win-shift, and so on with the exact same probabilities on each day as the actual animals but would only act upon these and ignore other behavioral options or task features, like the cue. This was contrasted with an “agent” that acted purely based on the 4 cue-related response options (“top-cue/left-response,” “top-cue/right-response,” “bottom-cue/left-response,” and “bottom-cue/right-response”), again with the probabilities for showing this behavior on each day instantiated by the animals’ actually displayed response frequencies. By running these agents for 1,000 iterations of the same basic task design and phases as used for the animals, bootstrap distributions were generated consistent with a pure outcome-based or pure cue-based strategy, applied with the same probabilities actually exhibited by the animals.

**Fig 5:**
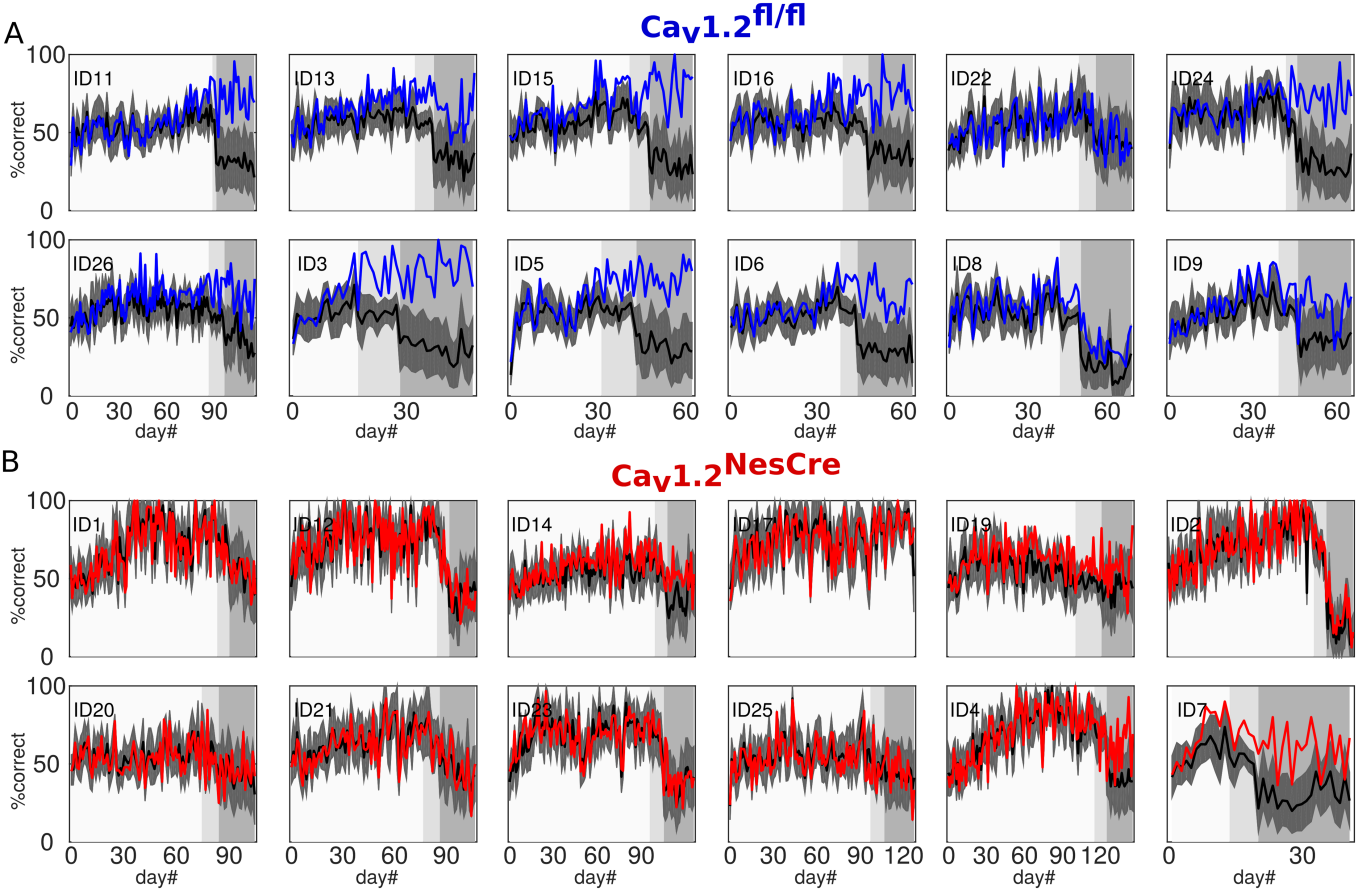
Outcome-rule-based bootstrap distributions for shift trials. A) Actual performance (blue curves) and bootstrapped performance distributions (gray-shaded: 90% CI, black: mean) generated from the purely outcome-based response behavior that is most consistent with the animal’s actual behavior, i.e., with day-specific outcome-rule choice probabilities inferred from the animal’s actual distribution of outcome-rule-consistent responses. Curves and corresponding bootstrap distributions are shown for all Ca_v_1.2^fl/fl^ animals. B) Same for Ca_v_1.2^NesCre^ animals. Note that while most Ca_v_1.2^fl/fl^ mice escape the bootstrap distributions as the task progresses (later in phase I or in phase II/III), most Ca_v_1.2^NesCre^ animals remain within the bootstrap 90%-confidence bounds. Data available at https://github.com/GKoppe/BehavioralData_Ana.

While 11/12 Ca_v_1.2^fl/fl^ animals escaped the outcome rule bootstrap distributions (i.e., performed significantly better) in both shift (Fig 5) and stay (S3 Fig) trials, this was only the case for 4/12 Ca_v_1.2^NesCre^ animals. Indeed, Ca_v_1.2^fl/fl^ and Ca_v_1.2^NesCre^ animals significantly differed with regards to their agreement with the outcome bootstraps (χ^2^ = 8.71, *p* = .003), indicating that performance in the Ca_v_1.2^NesCre^ group is much better explained in terms of adaptation of outcome-based strategies than is the case for Ca_v_1.2^fl/fl^ animals. Vice versa, when animal behavior was evaluated in terms of the cue rule bootstrap distribution, only 3/12 Ca_v_1.2^fl/fl^, but 10/12 Ca_v_1.2^NesCre^, animals escaped the cue rule distributions on either shift or stay trials, with, again, significant differences among the groups (χ^2^ = 8.22, *p* = .004, see S4 and S5 Figs). Thus, Ca_v_1.2^fl/fl^ animal behavior tended to be consistent with the cue rule but inconsistent with the outcome rules, while the reverse tended to be true for Ca_v_1.2^NesCre^ animals.

As a final step to prove that the observed performance progress across trials could indeed entirely be explained by adapting outcome- versus cue-based strategies, RL models were estimated from the behavioral data (see Materials and methods). In brief, these models consist of linear update rules for the values of (*situation*, *action*)-pairs (Eq 1 in Materials and methods) and a sigmoid-type choice probability function (Eq 3 in Materials and methods), which renders situation-dependent choices among response options, with probabilities based on the learned relative values of the different actions in that situation. The learning process itself was modeled by a Rescorla–Wagner (RW) rule [26], augmented by a Pearce–Hall (PH)-type “associability” mechanism [27], which regulates the learning rate in accordance with the recent history of reward prediction errors (see Materials and methods for details; a pure RW model and a simplified hybrid RW+PH model with 1 rate parameter fixed were checked as well and yielded almost identical results). Hence, these models would progress from trial to trial, sequentially gathering evidence for the different reward returns to be expected from the different response options in each environmental situation and issuing choices on each trial based on these learned values.

Two types of models were constructed, one equipped purely with the 4 elementary outcome rules (see above; termed “outcome model” in the following), ignoring the cue or any other potential strategy, and one with the 4 cue-based response options as given above (“cue model”), basing its choices solely on these. Thus, both models are described by exactly the same number of response options and parameters and differed solely in terms of what these response options would refer to. The set of 5 parameters that characterizes each of these models is comprised of a global learning rate *κ*, an additional time-dependent rate component regulated by a parameter *η* in accordance with the recent history of reward prediction errors, and 3 individual exploration parameters *β*_k_ for each separate task phase kϵ{I,II,III}. While the 2 learning rate parameters *κ* and *η* determine how values for (*situation*, *action*)-pairs are updated as the animals progress through their individual trial sequences (see Eq 2 in Materials and methods), the 3 parameters *β*_k_ control the steepness of the sigmoid choice function (Eq 3 in Materials and methods) and, thus, how deterministically (*β→∞*) or probabilistically (*β→*0) an animal would behave given the learned values. All parameters were estimated from the animal data by maximum likelihood, and models were compared via hierarchical Bayesian inference, yielding expected posterior probabilities for the 2 types of models (cue-based versus outcome-based) given the observed behavioral data (see Materials and methods for details).

Ca_v_1.2^NesCre^ animals were much better described by the RL model, which updates the 4 elementary outcome strategies, than by the cue RL model, with the expected posterior model probabilities being $\bar{p}(\text{outcome model}|\text{data})=.71$ versus $\bar{p}(\text{cue model}|\text{data})=.29$ (S6 Fig left) and an exceedance probability (i.e., the likelihood with which the posterior model probability of the outcome model exceeds that of the cue model; see Materials and methods) of *p* = .95 (S6 Fig right). By contrast, the behavior of the Ca_v_1.2^fl/fl^ animals was much better captured by the RL model updating cue-association strategies, with expected posteriors $\bar{p}(\text{cue model}|\text{data})=.79$ versus $\bar{p}(\text{outcome model}|\text{data})=.21$ and an exceedance probability for the cue over the outcome model of *p* = .99. This analysis confirms that an outcome model much more plausibly describes the behavior of the Ca_v_1.2^NesCre^ animals than a cue model, while, vice versa, the cue model agrees much better with the behavior of the Ca_v_1.2^fl/fl^ animals than the outcome model.

### Specificity to *Cacna1c* knockout

In our study, the specific *Cacna1c* knockout was achieved via the *Cre*-lox system using a Nestin promoter-driven Cre recombinase in mice double-transgenic for both Ca_v_1.2^fl/fl^ and Nestin-Cre (i.e., Ca_v_1.2^NesCre^). However, mice carrying the Nestin-Cre transgene alone (NesCre) have previously been associated with metabolic issues [28] as well as with potential deficits in learning [29], although that same study found that the deficit was rather specific to fear conditioning (freezing responses) and did not carry over to other kinds of learning. Nevertheless, given these previous indications, we conducted a further control study with 12 NesCre and 12 wild-type (WT) mice using the exact same experimental protocol as employed before. These 2 additional controls were run on experimental phase I only, since only the first task stage is required to assess whether the animals would pick up the cue rule in principle. As done above, we then compared all groups based on percentage of correct responses, bootstrap distributions designed to assess the deviation of actual performance from the one expected under a given response rule, and RL models. All of these 3 analysis approaches consistently indicated that the additional control groups are better described in terms of the cue-based response strategy: both NesCre and WT mice show increased accuracy (according to the cue rule) at the end of phase I compared to Ca_v_1.2^NesCre^ mice (ANOVA main effect on groups: *F*(3,44) = 15.04, *p <* .001; both post hoc tests *p <* .001, see also S7 Fig) but were not different from the previous Ca_v_1.2^fl/fl^ control group (both p > .99). Furthermore, only 1/12 NesCre and 0/12 WT mice left the 90% confidence bands of the cue rule bootstrap distribution, i.e., most animals showed performance levels as expected under the cue rule. Chi-square-based tests confirmed that significantly more of the NesCre and WT animals adhered to the cue rule than Ca_v_1.2^NesCre^ knockout animals (NesCre versus Ca_v_1.2^NesCre^: χ^2^ = 16.67, *p <* .001, WT versus Ca_v_1.2^NesCre^: χ^2^ = 20.31, *p <* .001), with numbers comparable to those within the original control group (NesCre versus Ca_v_1.2^fl/fl^: χ^2^ = 1.2, *p* = .273, WT versus Ca_v_1.2^fl/fl^: χ^2^ = 3.43, *p* = .064). Conversely, in terms of the outcome-based response rules, 11/12 NesCre and 10/12 WT mice went beyond the confidence bands of the outcome rule bootstrap distribution, i.e., significantly deviated from the performance expected under the outcome rule. Again, these numbers are significantly different from those in the Ca_v_1.2 knockout strain (NesCre versus Ca_v_1.2^NesCre^: χ^2^ = 8.71, *p* = .003, WT versus Ca_v_1.2^NesCre^: χ^2^ = 6.17, *p* = .013) but not from those in the Ca_v_1.2^fl/fl^ group (NesCre versus Ca_v_1.2^fl/fl^: χ^2^ = 0, *p* = 1, WT versus Ca_v_1.2^fl/fl^: χ^2^ = .38, *p* = .537; results were similar when only task phase I was considered for all animals, see Materials and methods). Lastly, our RL-model-based analysis revealed that, similar to the Ca_v_1.2^fl/fl^ controls, both NesCre and WT mice have a much higher posterior probability for the cue rule model (NesCre: $\bar{p}=.929$, WT: $\bar{p}=.929$) than for the outcome rule model (NesCre: $\bar{p}=.071$, WT: $\bar{p}=.071$). Thus, we conclude that the change in response strategy observed for the knockout strain is indeed specific to this group, that is, can be attributed to the ablation of Ca_v_1.2 calcium channels and is not due to other confounding factors.

## Discussion

In solving a specific cognitive task, animals may follow a variety of different behavioral strategies or ecological heuristics. Here, we demonstrate, through a detailed analysis of response patterns, reward returns, and computational models statistically estimated from the animals’ actual behavior [30], that both Ca_v_1.2^NesCre^ and Ca_v_1.2^fl/fl^ mice improve performance across different phases of a cue discrimination task but do so by fundamentally different means. While Ca_v_1.2^fl/fl^ mice seemed to infer the correct cue-response association, Ca_v_1.2^NesCre^ animals learned to increase their reward returns by conditioning their responses on previous outcomes in an optimal manner. It is thus important to note that while “standard” behavioral analysis would have inferred that both strains of animals, Ca_v_1.2^fl/fl^ and Ca_v_1.2^NesCre^, could basically learn the cue-discrimination rule, only with the Ca_v_1.2^NesCre^ animals being significantly worse than the Ca_v_1.2^fl/fl^, here we arrived at a quite different conclusion: in fact, our results suggest that Ca_v_1.2^NesCre^ animals did not learn the cue rule at all but rather based their behavior on adapting another reward-increasing strategy. We could also demonstrate that this alteration in behavioral strategy is specific to the Ca_v_1.2 ablation and cannot be attributed to the expression of Nestin-promoter driven Cre per se. That Ca_v_1.2^NesCre^ mice were still able to adapt their responses based on observed outcomes also demonstrates that Ca_v_1.2^NesCre^ mice do not suffer from a general learning deficit but perhaps one that is domain-specific or more closely tied to other cognitive capacities, like working memory [31].

### Behavioral strategies, heuristics, and learning as active decision making

In a real ecological context, there is a multitude of different factors and contextual conditions that will or could influence whether a behavioral plan will ultimately lead to success. When an animal hits on a favorable outcome it had not expected, it is often difficult to discern which of the many foregoing sensory inputs and actions were indeed crucial for predicting and achieving this success and which are irrelevant and would better be ignored; also termed the “credit assignment problem” [32]. To parse out the crucial and predictive factors from limited experience, the small sample of reality an animal has access to in its lifetime, would be difficult enough if the world were rather deterministic. But on top of that, the world is highly uncertain and probabilistic, full of unforeseen events that complicate inference on environmental structure. To deal with this, animals have evolved or acquired a larger repertoire of general strategies, heuristics, shortcuts, and response preferences [5,6] that could guide them through novel situations by biasing probability distributions toward certain subsets of situation/action/outcome triples and, potentially, are also designed to minimize an animal’s cognitive effort [33].

All these different response strategies and biases almost certainly come into play in any novel laboratory situation an animal faces. Animals are unlikely to behave completely randomly but, according to recent evidence, might actually actively probe and test out different such strategies and behavioral “hypotheses” whilst learning [12,13]. In rodents, common a priori strategies are win-stay, win-shift, lose-shift, or alternate [7,9,10], which all could make sense in one environmental setting or another [8,11,34]. For instance, rats hoard food in their underground burrows—once the food is gone from one of the chambers or, more generally, a food source is depleted, it is certainly reasonable to win-shift. In other circumstances, food sources may be expected to refill (perhaps in certain temporal intervals), such that a win-stay strategy would be more appropriate. Hence, it seems reasonable that even the Ca_v_1.2^fl/fl^ animals in our task reverted to one of these more basic strategies, namely win-stay, when the cue information became ambiguous on certain trials. In fact, some of the Ca_v_1.2^fl/fl^ animals that did not show as high performance levels as their group mates appeared to have adopted an outcome-based strategy as well, further illustrating that this is not a behavioral pattern “outside the normal scope” but a sensible, although suboptimal, way to approach this task.

Often, from an experimenter’s point of view, it might in fact not be that easy to construct experimental situations that clearly dissociate what the experimenter intended the animals to do from one of these basic, a priori strategies animals bring into the task. For instance, to probe working memory in rodents, delayed response or alternation tasks on a T-maze are often used. However, some studies have suggested that, rather than using working or short-term memory to encode correct responses as intended by the experimental design, rodents may rely on subtle external bodily cues like head orientation maintained throughout the delay phase of the task [14,15]. Such behavioral alternatives or confounds with other previously acquired or prepared strategies may easily be overlooked or, indeed, sometimes hard to avoid. There could also be other hidden dependencies or predictable structure in a task design (especially in learning tasks that evolve across trials) that are not immediately obvious and that animals may attempt to exploit. Even if overall (given unlimited experience) everything is well balanced and controlled by design, the individual history of trial drawings, responses, and outcomes may be locally highly nonrandom and bias animals differentially toward certain strategies rather than others [35, see also 36,37]. Thus, consideration of different possibly rewarding behavioral strategies may not only provide a lot more mechanistic insight by revealing the details of how an animal solves the task posed but may, in fact, prove important for drawing the right conclusions with respect to the involved cognitive and behavioral capacities.

For these reasons, it is also important that the behavioral analysis takes the animals’ perspective by taking into account the exact same set or sequence of trial types as encountered by the animals in order to accurately infer what they could have possibly learned about different behavioral policies given their individual choice and reward histories [35]. In fact, it is a particular advantage of the bootstrap- and model-based analyses reported here that they adopt the animal’s point of view, incorporating the exact same sequence of stimulus events, behavioral responses, and reward returns as experienced by the animals, and thus yield day-by-day (or even trial-to-trial) predictions on an individual animal basis (e.g., Fig 5). Further note that the next trial type encountered by an animal is often a consequence of the animal’s own behavior, as in the case of correction trials, hence a reflection of the animal’s own choices that should be acknowledged in an analysis of behavioral strategies. With regards to the specific experiments conducted here, we therefore would like to emphasize that none of the analyses made any a priori assumptions about the distribution or occurrences of trial types but went exactly with those trials as empirically observed and that, of course, the experimental conditions for all animal strains were exactly the same (and hence all differences in experienced trial sequences a consequence of differences in the animals’ behavior). It may also be worth noting that the animals showed strategy-consistent responses across all 3 task stages, although these considerably differed in the composition of trials and trial transitions they harbored.

### RL rules

Our RL model analysis was based on a Rescorla–Wagner rule augmented by a Pearce–Hall-type mechanism [27,38], in which the learning rate is regulated by the recent history of successful outcome predictions, increasing as the mismatches between predicted and experienced rewards become more numerous (and thus indicating to the animal that its current response policies need to be altered). Although the results using this type of learning rule were completely consistent with those obtained with a pure Rescorla–Wagner rule (as well as with a simplified hybrid model; see Materials and methods), we adopted this learning mechanism, as it turned out to yield the best description of the behavioral data based on Bayesian model comparisons (see S8 Fig). Animal learning has also variously been described in terms of stimulus-response versus action-outcome associations [39,40]. In light of this discussion, it may therefore be important to emphasize again that all animal strains studied here did indeed use the outcomes to regulate and advance their behavioral policies, i.e., were all able to utilize the reward feedback in adjusting their behavior in a profitable manner. The specific difference between the animal strains was that the Ca_v_1.2^NesCre^ mice did not base their responses on the presented cue but on where the reward occurred on the previous trial (i.e., on the previous outcome), and the bootstrap- and model-based analyses, which specify the assumptions about the behavioral process in formal detail, confirmed that this provided a sufficient explanation for the animals’ behavior.

### Calcium channels, learning, and clinical implications

Calcium (Ca^2+^) influx through *N*-methyl-D-aspartate (NMDA) and voltage-gated calcium channels is crucial for synaptic plasticity [24] and nuclear gene expression associated with plasticity [41] and cell survival [42]. Although there are several other sources of cellular Ca^2+^ influx, like NMDA or T- or N-type Ca^2+^ channels [43,44], a cortex-wide, functional ablation of L-type Ca^2+^ channels through a knockout of the *Cacna1c* gene is therefore supposed to impair plasticity, and thus learning, in a variety of settings. Indeed, this selective knockout has been associated with deficits in spatial memory [45,46] and observational fear learning [47]. Although the cortex-wide lack of Ca_v_1.2 in the present preparation prevents a more precise localization of the effects reported here, a recent study employing a similar touchscreen-based, two-choice stimulus discrimination task in mice revealed a specific dependence on dorsal striatum [48], in line with earlier studies linking this region to the mediation of SR associations [39,49,50]. Interestingly, this study also involved correction trials, which may facilitate outcome-based strategies, and found that performance scores in the lesioned animals settled around similar levels as observed for the Ca_v_1.2^NesCre^ mice here, about 60%–65%. Although outcome-based strategies were not investigated by Delotterie and colleagues [48], these findings could indicate that the present effects may be more specifically rooted in L-type Ca^2+^ channel dysfunction in dorsal striatum.

Findings like these may also open new perspectives on treatment options in psychiatry. Specifically, the *CACNA1C* gene has been associated with severe psychiatric illnesses such as depression [51], bipolar disorder [52], autism [53], and schizophrenia [51,54], for which it has been listed as a risk gene [51,55]. The present results suggest that for modifying a patient’s behavior, certain avenues, potentially resting on biologically more elementary behavioral options, may be fruitful to explore where other forms of behavioral therapy have failed. They at least suggest that a detailed scrutiny of how a patient solves a specific set of cognitive or emotional tasks, on top of standard clinical assessment, may provide valuable insights into how to best address the “behavioral malfunctioning” of a patient in terms of the specific behavioral interventions and rules the therapy should focus on (see also [56]). Hence, in future applications, the combination of standard cognitive test batteries with model-estimation techniques, as used here, could lead into the design of more specifically targeted, individualized behavioral therapies, on top of its potential use for diagnosis and prediction.

## Materials and methods

### Ethics statement

All experiments complied with regulations covering animal experimentation within the EU (European Communities Council Directive 2010/63/EU), and were approved by German animal welfare authorities (Regierungspraesidium Karlsruhe: ethical approval no. 35-9185-81-G-227-12).

### Animals

All mice were bred in the animal facility of the Central Institute of Mental Health, Mannheim, Germany, and maintained on a C57BL/6N background. CNS-specific ablation of the L-type voltage gated calcium channel Ca_v_1.2 was achieved by inactivation of the *Cacna1c* gene using the cre-loxP system. More specifically, Ca_v_1.2^NesCre^ mice (genotype: Ca_v_1.2 L2/L2, Nestin-Cre -/+) were generated by crossbreeding mice carrying 2 loxP-flanked (“floxed”) Ca_v_1.2 alleles [45] and mice with an additional heterozygous transgene expressing Cre recombinase under the control of a Nestin promoter [57]. In these animals, the loss of Ca_v_1.2 functionality is accomplished by the excision of *Cacna1c* exons coding for the IIS5 and IIS6 transmembrane segments of the pore-forming subunit α1C of the *Cacna1c* gene via Cre recombinase that is expressed in all cell types of the CNS through embryonic development. Crossbred Ca_v_1.2^fl/fl^ mice homozygous for the floxed Ca_v_1.2 allele but lacking Cre recombinase expression (genotype: Ca_v_1.2 L2/L2, Nestin-Cre -/-), and thus not suffering calcium channel loss, served as primary controls within the experiment. To rule out that observed learning differences between these 2 groups were not attributable to metabolic [28] or other potential factors [29] associated with the expression of Cre recombinase in the Ca_v_1.2^NesCre^ group, we assessed performance of 2 additional control groups: animals with a heterozygous transgene expressing Cre recombinase under the control of a Nestin promoter without loxP-flanked Ca_v_1.2 alleles (NesCre; genotype: Ca_v_1.2 +/+, Nestin-Cre +/-) and pure wild-type animals (WT; genotype: Ca_v_1.2 +/+, Nestin-Cre -/-).

In total, 12 male Ca_v_1.2^NesCre^, 12 Ca_v_1.2^fl/fl^, 12 NesCre, and 12 WT mice were single-housed in conventional macrolon cages (Type II, 26 × 20 × 14 cm) with sawdust (RehofixMK-2000; Rettenmaier & Söhne, Rosenberg, Germany), nesting material, and free access to water. Single housing was chosen as it has been shown to be less stressful for male mice than group housing under standard maintenance conditions (i.e., no enrichment) [58]. All animals were approximately 12 weeks old at experimental onset. Colony room settings included a temperature of 23±2°C, relative humidity 50%± 5%, and a reversed 12 hour light–dark schedule with the lights off at 7:00 AM [58,59]. Experiments were conducted during the dark phase, which constitutes the active phase of mice.

Mice were food restricted [60] to 85%–90% of their initial free-feeding body weight in order to maintain a high degree of motivation during operant training. The mean initial body weight was assessed on 5 consecutive days when animals had free access to food. Body weight and health status were monitored daily prior to testing. At the start of food restriction, mice were food deprived for 1 light phase and received 2.0 g to 3.3 g food in the subsequent dark phase, depending on individual loss. Henceforth, the amount of food was adjusted in accordance with the deviation from the intended 87.5% of initial body weight. Touchscreen-trained mice additionally received 7 μl sweet condensed milk (SCM; Milchmaedchen, Nestle, diluted in 1:4 tap water) as a reward for correct responses during training. They were previously acquainted to SCM in their home cages to avoid later refusal as a reward. In order to minimize handling as a source of anxiety and to reduce stress, all mice were handled without physical restraint following the cup handling protocol described in [61]. Mice were scooped up and allowed to walk freely over the handler’s open hands.

### Stimuli and apparatus

Mouse touchscreen chambers (Model 80614–20, Campden Instruments Ltd., Loughborough, Leics., United Kingdom) included several infrared (IR) light beams for movement detection, a 3 W house light for controllable illumination, and a tone generator for auditory signaling. The inner chamber consisted of black Perspex walls arranged in a trapezoid shape (height 19 cm, width 24 respectively 6 cm, depth 17 cm) and a metal grid floor. The longer end of the chamber was equipped with a touch-sensitive screen (IR-detector-based) partly covered by a 3-hole Perspex mask in order to separate the display into three equal response windows (7 x 7 cm). The lateral fields were used to detect touches, while the central field was used to display the reward-side-indicating cue. Reward cues consisted of bright gray 2.5 cm squares (brightness = 70%, 85%, or 96%, hue and saturation = 0 in HSB colors). Correct responses triggered the display of a tone, illumination in the reward tray, and delivery of 7 μl sweet condensed milk. The reward tray (2 x 2 x 2 cm) of an externally placed feeder for liquid suspensions positioned at the touchscreen-opposed end of the chamber contained another light beam detector used to count entries, start and stop latencies, and trigger initiation of the next trial. Hence, the subject was required to initiate a trial at the narrow end of the chamber, subsequently traverse to the opposed touchscreen side for response, and, finally, traverse back in order to retrieve the reward. This ensured optimal prospect to the screen and constant start conditions on each trial.

### Experimental procedure

#### Preliminaries, familiarization, and habituation

The experimental paradigm followed a fixed daily schedule in which mice were transported to the test room, acclimatized, and introduced to the touchscreen chambers. Body weight was monitored and test chambers were cleaned with desalinated water before entrance. Animals were first habituated and familiarized with the experimental setup before transferal to 3 consecutive test phases (labeled I–III), which varied in their composition of trial types (see below).

Habituation procedures were carried out as described in detail in Talpos and colleagues [62]. Briefly, mice became acquainted to the setup, procedures, and the basic functions of the chamber. Habituation included stepwise presentation of principal functions in the chamber: SCM delivery associated with a signaling tone, touching the lateral fields of the screen in order to receive a reward, triggering the next trial self-contained by head entries into the feeder after a 5 s intertrial interval (ITI), and time out with house light illumination as a signal for incorrect responses. Time spent in the chamber was increased stepwise from 10 to 40 min, and an initial phase of free milk supply was added, which had to be triggered by nose poke responses on the touchscreen.

Familiarization procedures were carried out as described in detail in Richter and colleagues [63]. Mice were first familiarized with the fact that touching 1 of the 2 lateral touch fields on which a cross was displayed would yield reward. After accomplishing 30 touches to the lateral touch fields within 30 min, mice were introduced to the reward-indicating cue on the cue presentation field and were for the first time confronted with the basic cue rule, which requires the animals to associate a cue position (top, bottom) with a response side (left, right). Correct responses were rewarded with delivery of SCM, but, different from the proper test phases, touching the incorrect touch field did not result in a time-out. The session was terminated after 60 min or when 40 trials were completed. Animals progressed to the experimental test phases after completing 40 trials on 2 consecutive days.

#### Experimental task phases

The experiment consisted of 3 successive test phases. While Ca_v_1.2^NesCre^ and Ca_v_1.2^fl/fl^ underwent this whole experimental protocol, the additional controls, NesCre and WT mice, were tested only on phase I (see last Results section and below). The 3 test phases varied in their composition of different trial types (see Fig 1), with additional probe trials (see below) inserted in stages II and III. This was done only in consecutive task stages and not all at once, as otherwise, it would have made the task too complicated for the animals and impeded learning in general. The 3 test phases also varied in the relative frequencies of stay and shift trials (see S1 Fig), allowing for a more thorough assessment of strategy adaptations, wherein a stay (shift) trial was defined here as a trial in which, for obtaining a reward, the response side would have to be maintained or switched, respectively, depending on whether the previously rewarded (i.e., correct) response side was the same (stay) or the opposite one (shift). Incorrect responses according to the experimenter-defined cue rule were punished by a 5 s timeout and house light presentation.

Phase I consisted of high-contrast stay or shift trials in which only the reward-indicating cue (bright gray square) was presented on the screen. After incorrect responses, correction trials were inserted to facilitate learning, i.e., cues were repeatedly presented in the same position until the correct response was performed (effectively making lose-shift reward probability 1 in these trials). Following a correct response, stay and shift trials were drawn with equal probability. In case the reward-indicating cue was presented on the same position 3 times in a row, a shift of cue position was enforced (to discourage a pure side bias but thus slightly favoring shift responses). A session (per day) was completed after 40 trials or 60 min. In order to progress to phase II, animals had to achieve ≥ 80% accuracy on 2 consecutive days. However, progression into phase II was enforced for all remaining mice when the least-advanced animals had completed at least 70 sessions of phase I. Note that 1 Ca_v_1.2^NesCre^ animal died after 104 days of testing and thus dropped out of analyses for phase II and III.

In phase II, shift trials with lowered stimulus contrast were introduced, in which a second, less salient cue (with reduced brightness = 20%) was presented in the position opposite to the target cue (brightness = 85%, i.e., top if the reward-indicating cue was presented at the bottom and vice versa; see Fig 1II/1III). It was reasoned that such trials with reduced cue saliency would make it harder to apply the cue rule and thus provide an additional test which should affect cue- but not outcome-based responses. As in phase I, correction trials were presented to facilitate cue rule learning. Also, shift trials were always followed by a minimum of 2 (high-contrast) stay trials (encouraging outcome-based win-stay responses). Subsequently, stay trials were drawn with probability .5, with high- and low-contrast shift trials with *p* = .25 each. A session was completed after 50 trials or 60 min. Progression to phase III was again approved after ≥ 80% accuracy on 2 consecutive days. Animals which had not fulfilled phase I progression criteria were trained for 14 days before transiting into phase III.

In phase III, in addition to the trial types present in phase II, ambiguous trials with 2 equally bright cues (brightness = 50%) were introduced. Again, these served as additional probes for potential cognitive biases or strategies endorsed by the animals (including the tendency to perseverate or alternate)—essentially, this type of manipulation should only influence cue- but not outcome-based strategies. In contrast to the other test phases, however, errors did not trigger a correction trial in phase III. Similar to phase II, a slightly higher probability for stay trials remained due to the fact that a (high-contrast) stay trial was inserted after every other trial. The subsequent probability for drawing a (high-contrast) stay trial was .5 and .125 for all other trial types. Phase III consisted of 20 consecutive days of testing with 100 trials per day. In total, given differences in learning speed, Ca_v_1.2^NesCre^ mice were tested for an average of 114 +/- 28 days, and Ca_v_1.2^fl/fl^ for 78+/- 19.

Potential side biases were evaluated in the Ca_v_1.2^NesCre^ and the Ca_v_1.2^fl/fl^ mice on the first day of experimental testing (in phase I), but neither did the proportion of left-side responses in either group significantly depart from chance (Ca_v_1.2^NesCre^: *t*(11) = –.748, *p* = .47; Ca_v_1.2^fl/fl^: *t*(11) = .647, *p* = .53) nor was there a significant difference between the two groups (independent *t* test for Ca_v_1.2^NesCre^ versus Ca_v_1.2^fl/fl^: *t*(22) = .981, *p* = .337). Additional tests with an artificial agent (see below), acting according to different probabilities for left and right responses throughout all experimental phases (varying probability for left responses from .1 to .9), further revealed that any such side bias would not have significantly altered the perceived reward for different outcome-based strategies. Hence, we conclude that even if a side bias had been present, it would not have affected learning of particular strategies.

### Data analysis

#### Cue and outcome rule performance

Performance according to the cue rule was quantified in terms of the (relative) number of correct (rewarded) responses on a given day or phase. To assess whether performance deviated from chance individually for each subject and task phase, the binomial distribution *B*(K,N,p), with parameters *p* = 0.5, N = total number of trials, and K = number of correct cue rule response trials, was employed. At the group level, in addition, one-sample *t* tests were performed for each group on the last day of each phase, and on the last versus first day of phase I, to test the relative number of correct responses against chance and against performance at outset. For assessing overall group and task phase differences, 2 × 3-factorial ANOVAs were conducted with the between-subject factor “group” (Ca_v_1.2^fl/fl^ versus Ca_v_1.2^NesCre^) and repeated measurement factor “task phase” (I-III). Analyses were run either across all trials or separately for stay and shift trials. As low-contrast trials were introduced only in experimental phases II and III and could thus not be entered as separate factors into the ANOVA, performance was collapsed over high- and low-contrast trials for this type of analysis. Degrees of freedom were Greenhouse–Geisser-corrected in case of sphericity violations. Post hoc pairwise comparisons were conducted based on the estimated marginal means and Bonferroni-corrected for multiple comparisons. Low-contrast trials, which (unlike high-contrast trials) only occurred in phases II and III, were separately analyzed via dependent *t* tests for these 2 task phases for each group.

Win-stay, win-shift, lose-stay, and lose-shift responses, commonly denoted as “outcome rules” here (as opposed to the experimentally defined operant “cue rule”), were quantified in terms of the relative number of responses to the same (stay) or opposite (shift) side with respect to that of the previous response on previously correct (win) or incorrect (lose) trials, respectively, where “relative” means with respect to the total number of correct (win) or incorrect (lose) responses. Evidence indicates that these 4 response components are updated independently [7,64–66], potentially even mediated by different structures [66–68], such that a separate analysis of these 4 rules was favored here. This also facilitated comparison with the cue rule, which also comprises 4 elementary response options (“top cue–left response,” “bottom cue–left response,” “top cue–right response,” “bottom cue–right response”). Occurrence rates of these outcome rules were then compared via ANOVAs, dependent *t* tests for ambiguous versus nonambiguous trials in phase III, and binomial tests, which were used to test against chance for single subject analysis. Note that by symmetry, the probability for win-stay responses will be exactly 1-win-shift (and lose-stay = 1-lose-shift), such that only 2 outcome rules are independent and needed to be entered into the statistical design. Thus, a 2 (group) x 3 (task phase) x 2 (outcome rule: win-shift, lose-shift) ANOVA was conducted, with Bonferroni-corrected post hoc pairwise tests. Win-stay occurrence rates to ambiguous and nonambiguous trials were further correlated across days in phase III, wherein the Fisher transformation was applied to the (Pearson) correlation coefficients to make their distribution (approximately) normal and allow for comparisons via dependent one-sample *t* tests and two-sample *t* tests.

#### Reward probabilities associated with outcome rules

Reward probabilities for each outcome strategy per day were defined as the numbers of times a rule was reinforced divided by the total numbers of times it was applied. Analyses of reward probabilities were conducted on the Ca_v_1.2^NesCre^ animals via a 3 (phase) x 4 (outcome rules: win-stay, win-shift, lose-stay, lose-shift) ANOVA. Note that in this case, all 4 outcome rules are entered into the analysis since, different from the analysis of frequency of rule application described above, the reward probabilities for contrasting response pairs (i.e., lose/win shift versus stay) are not complementary (i.e., do not necessarily add up to 1) across the empirically encountered series of trials. This is because, on any given trial, the reward probability for the response not chosen is not subjectively available to the animal, i.e., shift versus stay probabilities are evaluated on different sets of trials, since we were interested in how rewarding a given outcome rule could have been perceived from the animal’s perspective. Where appropriate, group analyses were complemented by single-subject binomial tests on number of rewards obtained against chance.

#### Outcome versus cue rule bootstrap distributions

To explicitly test the null hypothesis that performance in each animal could be entirely accounted for by adaptation of the outcome rules or the cue rule SR pairs, we constructed a bootstrap distribution generated by “artificial agents” faced with the same experimental task structure, sampling actions on an equal number of days and trials as each animal. The outcome rule agent sampled actions from win-stay, win-shift, lose-stay, and lose-shift strategies with the same average frequencies observed for each animal per day. For instance, if an animal showed 42% win-shift versus 58% win-stay behavior on day 10, then after each correct (win) trial on day 10, the agent would sample its next response with these same probabilities. The cue rule agent sampled actions based on “cue-response” probabilities, namely “top-cue/left-response,” “top-cue/right-response,” “bottom-cue/left response,” and “bottom-cue/right response,” with response probabilities for each of these 4 pairs further split according to whether an animal faced a low- or a high-contrast trial. This procedure ensures that the agent’s responses on each different trial type follow as closely as possible the animal’s actual behavior, except—potentially—for the only 1 crucial aspect that the choice probabilities otherwise depended only on either the cue but not the previous outcome (cue rule) or vice versa (outcome rule).

Note that, in essence, for these types of bootstraps, responses are simply drawn from a set of defined available options with the same day-, animal-, and trial-type-specific response probabilities as empirically observed (inferred) (that is, this type of analysis does not refer to a true RL agent as described further below). This bootstrap distribution thus captures the pure scenario of solely outcome-based responses that is still most consistent with the animals’ actual behavior. Hence, if the animal performance lies within the confidence limits of this distribution, one would have to accept the H_0_ that outcome-based response strategies suffice to produce the observed performance levels without applying the cue rule (and vice versa, for the cue-rule-based bootstrap distribution).

For each animal, a total of 1,000 cue-/outcome-rule bootstrap replications were produced, from which the bootstrapped 90% confidence intervals were determined [e.g. 69]. Based on these, the number of days in which the animal’s performance lay outside this confidence region on either shift or stay trials was obtained, and the overall probability for this event given the total number of tests (days) performed was assessed via a binomial distribution with probability parameter α = .05. To yield an overall statistical assessment of whether Ca_v_1.2^NesCre^ and Ca_v_1.2^fl/fl^ animals differed with respect to overall rule-consistent behavior, the numbers of animals performing significantly better than the agent were then determined for each group and compared using chi-square tests. Binomial tests were also conducted within each group to determine whether the number of animals that escaped the bootstrap distributions may have been expected by chance given the *α* = .05 significance level.

For completeness, in addition to the results reported in the last Results section, we here note that similar differences between the Ca_v_1.2^NesCre^ and all other experimental groups were obtained when only task phase I was entered into the analysis (consistency with outcome-rule bootstrap distribution: Ca_v_1.2^NesCre^ versus NesCre: χ^2^ = 10.97, *p <* 0.001; Ca_v_1.2^NesCre^ versus WT: χ^2^ = 8.22, *p* = 0.004; Ca_v_1.2^fl/fl^ versus NesCre: χ^2^ = 1.2, *p* = 0.273; Ca_v_1.2^fl/fl^ versus WT: χ^2^ = 0.25, *p* = 0.615; consistency with cue rule bootstrap distribution: Ca_v_1.2^NesCre^ versus NesCre: χ^2^ = 8.71, *p* = 0.003; Ca_v_1.2^NesCre^ versus WT: χ^2^ = 12, *p <* 0.001; Ca_v_1.2^fl/fl^ versus NesCre: χ^2^ = 0, *p* = 1; Ca_v_1.2^fl/fl^ versus WT: χ^2^ = 1.04, *p* = 0.307).

### Computational modeling

#### RL models

Two RL models were set up, one implementing cue-rule and the other outcome-rule learning, to formally test hypotheses about the involved learning processes through model comparison. RL models consist of updating rules for the values *V*_t_ of state-action pairs (*s*,*a*), formalized here through a hybrid Rescorla–Wagner/ Pearce–Hall update mechanism [27,70] given by

$$\;V_{t+1}\left(s,a\right)=V_{t}\left(s,a\right)+\kappa \cdot \alpha_{t}\cdot \delta ,\;with\;\delta =r_{t}-V_{t}\left(s,a\right)$$ (1)

where *δ* is the reward prediction error, i.e., the difference between the actually obtained reward *r*_*t*_ and the expected reward value *V*_*t*_*(s*,*a)* for action *a* in situation *s*, *κ* is a constant global learning rate parameter, and *α*_t_ represents a time-dependent rate component which adjusts in accordance with the average accuracy of recent predictions, specified through a stable autoregressive process with the prediction errors *δ* as deterministic (external) inputs [70]. Specifically, *α*_*t*_ increases with the absolute size of the current prediction error and decays exponentially in time otherwise (with the tradeoff between these 2 processes regulated by a parameter *η*):

$$\alpha_{t}=\left(1-\eta \right)\cdot \alpha_{t-1}+\eta \cdot \left|\delta \right|,\;\eta \in \left\{0,1\right\}$$ (2)

As parameters *κ* and *η* might be partly correlated (given that they multiply), reduced models with *κ* = 1 fixed were run as well, as were models with the learning process given by a pure Rescorla–Wagner rule. While results using all 3 learning mechanisms were the same, the full hybrid RW+PH learning model was adopted here for presentation in the main text since it yielded the highest posterior probabilities in Bayesian model comparisons (see below; S8 Fig).

Learned values are then translated into action probabilities via a softmax function:

$$p\left(a|s\right)=\frac{e^{\beta_{k}\cdot V_{t}\left(s,a\right)}}{\sum_{l}e^{\beta_{k}\cdot V_{t}\left(s,l\right)}}\;,$$ (3)

where the *β*_*k*_ are parameters governing the exploitation/exploration trade-off in the 3 experimental phases indexed by k [71]. A high *β* means that the animal more strongly exploits the most-rewarding actions, while a low *β* produces more random or explorative behavior.

The “cue model” describes learning in terms of the operant cue rule only, i.e., only values for the 4 state-action pairs (“top-cue/left-response,” “top-cue/right-response,” “bottom-cue/left-response,” and “bottom-cue/right-response”) were tracked. The “outcome model,” by contrast, describes learning in terms of the outcome rules only, i.e., only values for the 4 state-action pairs “win-stay,” “win-shift,” “lose-stay,” and “lose-shift” were tracked.

#### Model estimation and selection

Given the action probabilities as defined by Eq 3, which incorporates the history of previous situations, actions, and outcomes through the model’s value function *V*_*t*_*(s*,*a)* (Eq 1), the model log-likelihood can be expressed as *l* = *Σ*_t_log*p*(*a*_t_|*s*_t_,*V*_t_,***θ***). Model parameters ***θ*** *=* {*κ*, *η*, *β*_*I*_, *β*_*II*_, *β*_*III*_}, where *κ* ϵ {0, 1} and *β*_*k*_ >0, were estimated for each animal by maximum likelihood using MATLAB’s active set algorithm (fmincon), which relies on solving the Karush–Kuhn–Tucker equations by quasi-Newton methods, starting from 100 different initial conditions to avoid local minima. The maximized log-likelihood value was later entered into a model comparison analysis (see below).

In order to determine which model best accounted for the observed behavior, we conducted Bayesian model selection (BMS) for group studies [72] on both groups separately. BMS is a hierarchical Bayesian framework which, at the top level, specifies a distribution across model probabilities *π*_m_ given the data, naturally expressed (since we are dealing with categorically distinct models) as a Dirichlet distribution (which reduces to the beta distribution in our two-model case). Based on this distribution, with parameters ***α*** estimated from the behavioral data, one can compute exceedance probabilities as $p(\pi_{m}>\pi_{m\prime }|\alpha ,\text{X})=p(\pi_{m}>.5|\alpha ,\text{X})$ given the data **X**, i.e., the likelihood with which the probability for model *m* exceeds that of model *m*’ (or 0.5 for a two-model comparison), as shown in S6B Fig. Likewise, we can infer the expected posterior model probabilities $\bar{p}(m|\text{X}):=E\left[\pi_{m}\right]$ as depicted in S6A Fig. Thus, rather than assuming each group to strictly follow one model (i.e., a fixed effect), this approach allows one to take into account variability between subjects within each group (i.e., random effects), as both Ca_v_1.2^NesCre^ and Ca_v_1.2^fl/fl^ animals may in principle follow either one or the other rule. Log-model-evidences *p*(**X** | *m*) as needed for the computation of the posteriors were approximated by the Bayesian Information Criterion ([73]; note, however, that in our case, the number of parameters was exactly the same for both models). Thus, for both groups, this approach evaluates whether the animals’ behavior is better described in terms of pure cue-rule versus pure outcome-rule learning through estimation of the posterior probabilities for the respective models given the animals’ actually observed sequence of behavioral choices.

All statistical analyses were conducted using IBM SPSS Statistics Version 22 (IBM Corp., 2013, Armonk, NY) and self-written MATLAB (Mathworks Inc., Sherborn, MA) scripts.

## Supporting information

S1 Fig Transition probabilities for different trial types in the three experimental phases. In phase I and II, green and red lines indicate transitions from correct and incorrect choices, respectively. Blue outlines mark newly introduced trial types in phases II and III. HC = high contrast; LC = low contrast.
(TIF)

S2 Fig Correlation between propensity to show outcome-rule consistent behavior and reward success. A) Overlaid overall percentage of shift-responses (red) and reward feedbacks on shift trials (magenta) across days and task phases for Ca_v_1.2^NesCre^ animals. Error shadings = SEM. B) Same for Ca_v_1.2^fl/fl^ animals. C) Mean percentage of shift-responses against reward on shift-trials for Ca_v_1.2^NesCre^ (red) and Ca_v_1.2^fl/fl^ (blue) animals. While for Ca_v_1.2^NesCre^ animals there is a highly significant correlation between the propensity to shift and reward feedbacks received (*r* = .93, *p* < .001), this is not the case for Ca_v_1.2^fl/fl^ animals (*r* = .08, *p* = .339). D) Same as A for stay responses and reward success on stay trials. E) Same as B for stay responses and reward success on stay trials. F) Same as C for stay responses and reward success on stay trials. Both groups exhibit a significant correlation between stay frequency and reward success on stay trials (Ca_v_1.2^NesCre^: *r* = .63, *p* < .001, Ca_v_1.2^fl/fl^: *r* = .33, *p* = .001). The two distinct clusters apparent for Ca_v_1.2^NesCre^ animals reflect stay response–reward correlations in different task phases. Data available at https://github.com/GKoppe/BehavioralData_Ana.
(TIF)

S3 Fig Outcome-rule bootstrap distributions for stay trials. A) Actual performance (blue curves) and bootstrapped performance distributions (gray-shaded: 90% CI, black: mean) generated from the purely outcome-based response behavior that is most consistent with the animal’s actual behavior, i.e. with day-specific outcome-rule choice probabilities inferred from the animal’s actual distribution of outcome-rule-consistent responses. Curves and corresponding bootstrap distributions are shown for all Ca_v_1.2^fl/fl^ animals. B) Same for Ca_v_1.2^NesCre^ animals. Note that while most Ca_v_1.2^fl/fl^ mice escape the bootstrap distributions as the task progresses (later in phase I, or in phase II/III), most Ca_v_1.2^NesCre^ animals remain within the bootstrap 90%-confidence bounds. Data available at https://github.com/GKoppe/BehavioralData_Ana.
(TIF)

S4 Fig Cue-rule based bootstrap distributions for shift trials. A) Actual performance (blue curves) and bootstrapped performance distributions (gray-shaded: 90% CI, black: mean) generated from the purely cue-based response behavior that is most consistent with the animal’s actual behavior, i.e. with day-specific cue-rule choice probabilities inferred from the animal’s actual distribution of cue-rule-consistent responses. Curves and corresponding bootstrap distributions are shown for all Ca_v_1.2^fl/fl^ animals. B) Ca_v_1.2^NesCre^. Data available at https://github.com/GKoppe/BehavioralData_Ana.
(TIF)

S5 Fig Cue-rule bootstrap distribution for stay trials. A) Actual performance (blue curves) and bootstrapped performance distributions (gray-shaded: 90% CI, black: mean) generated from the purely cue-based response behavior that is most consistent with the animal’s actual behavior, i.e. with day-specific cue-rule choice probabilities inferred from the animal’s actual distribution of cue-rule-consistent responses. Curves and corresponding bootstrap distributions are shown for all Ca_v_1.2^fl/fl^ animals. B) Same for Ca_v_1.2^NesCre^. Data available at https://github.com/GKoppe/BehavioralData_Ana.
(TIF)

S6 Fig Bayesian comparison of cue-rule vs. outcome-rule reinforcement learning (RL) models inferred from the animals’ actual behavior. A) Expected posterior probabilities for cue and outcome model given Ca_v_1.2^NesCre^ and Ca_v_1.2^fl/fl^ data. B) Exceedance probabilities (i.e., the probabilities with which the posterior model probability of one model exceeds that of the other).
(TIF)

S7 Fig Cue-rule consistent (i.e., correct) responses across sessions for NesCre and WT animals. Mean and SEM of the percentage of correct responses by session for NesCre (yellow) and wild-type (black) mice for (A) stay trials, and (B) shift trials. In contrast to Cav1.2^NesCre^ mice, both control groups exhibit a clear and significant improvement on stay trials during experimental phase I. On average, WT animals required 39.6 (+/- 11.5) and NesCre animals 40.3 (+/- 13.3) sessions to reach criterion. Data available at https://github.com/GKoppe/BehavioralData_Ana.
(TIF)

S8 Fig Bayesian comparison of models with different update rules estimated from the animals’ behavior. Expected posterior probabilities for a pure Rescorla-Wagner model, a hybrid model with a fixed global learning constant, and a hybrid model with the global learning rate parameter estimated from the data (see Materials and methods). Log model evidences that went into the model comparison were computed by averaging the BIC values across the cue and outcome models for each update rule.
(TIF)

## Abbreviations

BMS: Bayesian model selection
IR: infrared
ITI: intertrial interval
KO: knockout
NMDA: *N*-methyl-D-aspartate
PH: Pearce–Hall
RL: reinforcement learning
RW: Rescorla–Wagner
SCM: sweet condensed milk
SR: stimulus-response
WT: wild-type
