# Supplementary figures and images for: CACNA1C gene regulates behavioral strategies in operant rule learning
Source: PLoS Biol. 2017 Jun 12;15(6):e2000936. doi: 10.1371/journal.pbio.2000936 (PMC5467799; doi:10.1371/journal.pbio.2000936)

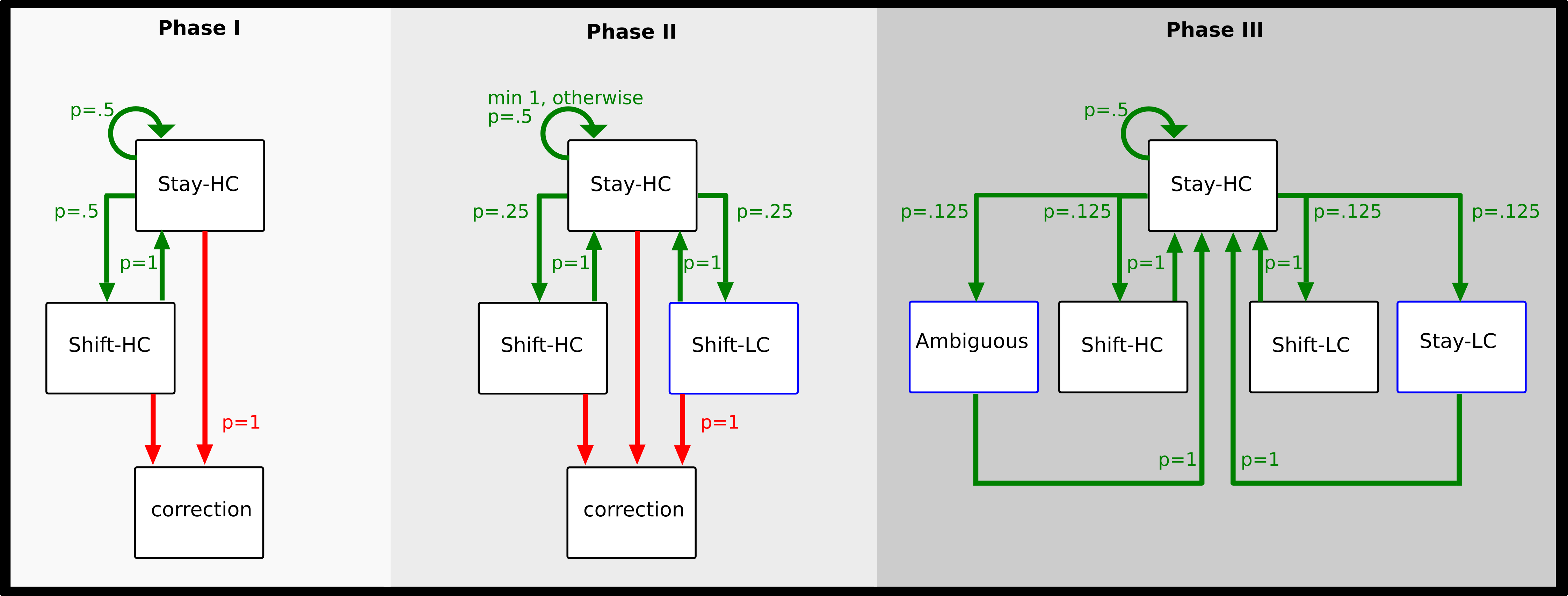

Supplement: S1 Fig — In phase I and II, green and red lines indicate transitions from correct and incorrect choices, respectively. Blue outlines mark newly introduced trial types in phases II and III. HC = high contrast; LC = low contrast. (TIF) [file pbio.2000936.s001.tif]

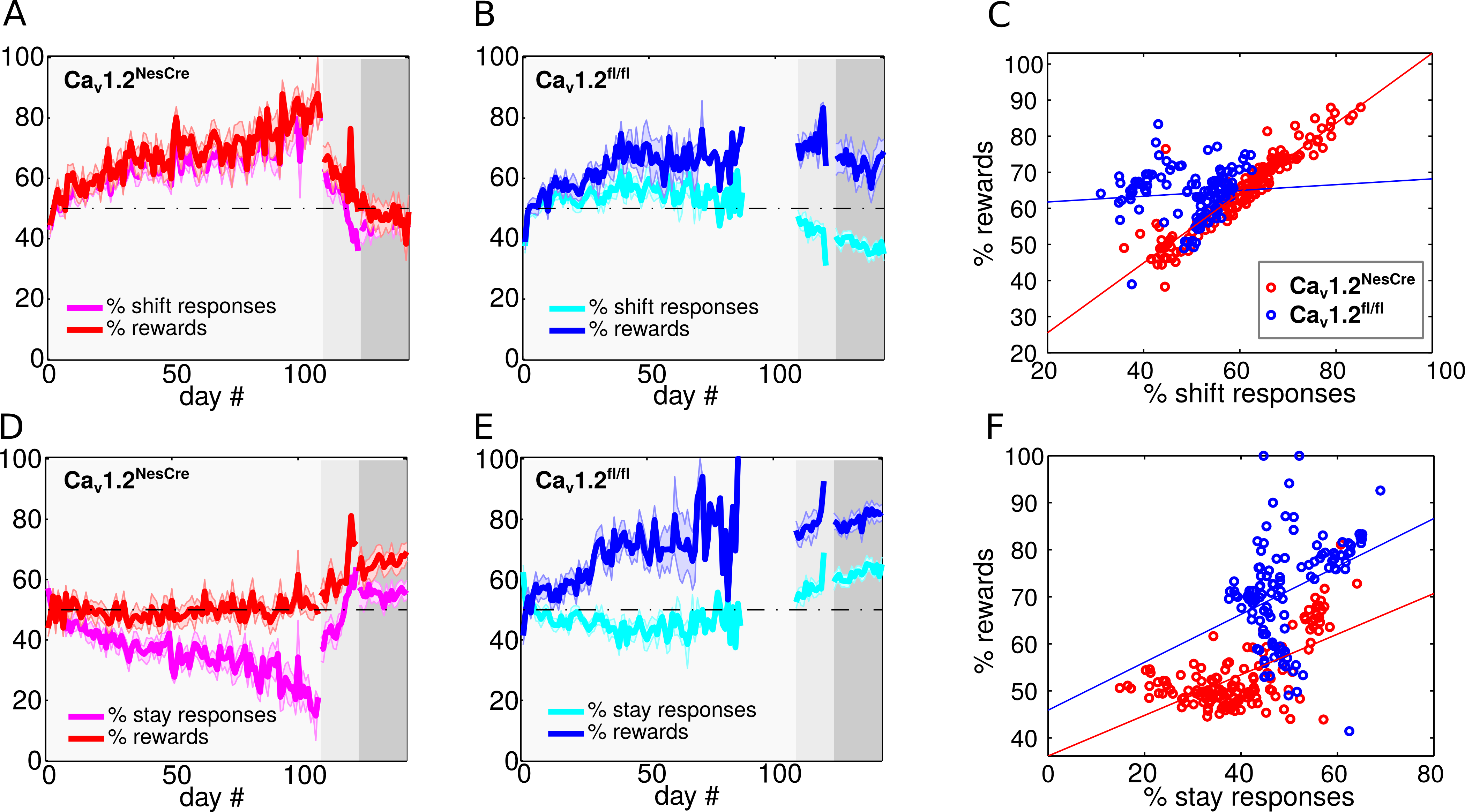

Supplement: S2 Fig — A) Overlaid overall percentage of shift-responses (red) and reward feedbacks on shift trials (magenta) across days and task phases for Cav1.2NesCre animals. Error shadings = SEM. B) Same for Cav1.2fl/fl animals. C) Mean percentage of shift-responses against reward on shift-trials for Cav1.2NesCre (red) and Cav1.2fl/fl (blue) animals. While for Cav1.2NesCre animals there is a highly significant correlation between the propensity to shift and reward feedbacks received (r = .93, p < .001), this is not the case for Cav1.2fl/fl animals (r = .08, p = .339). D) Same as A for stay responses and reward success on stay trials. E) Same as B for stay responses and reward success on stay trials. F) Same as C for stay responses and reward success on stay trials. Both groups exhibit a significant correlation between stay frequency and reward success on stay trials (Cav1.2NesCre: r = .63, p < .001, Cav1.2fl/fl: r = .33, p = .001). The two distinct clusters apparent for Cav1.2NesCre animals reflect stay response–reward correlations in different task phases. Data available at https://github.com/GKoppe/BehavioralData_Ana. (TIF) [file pbio.2000936.s002.tif]

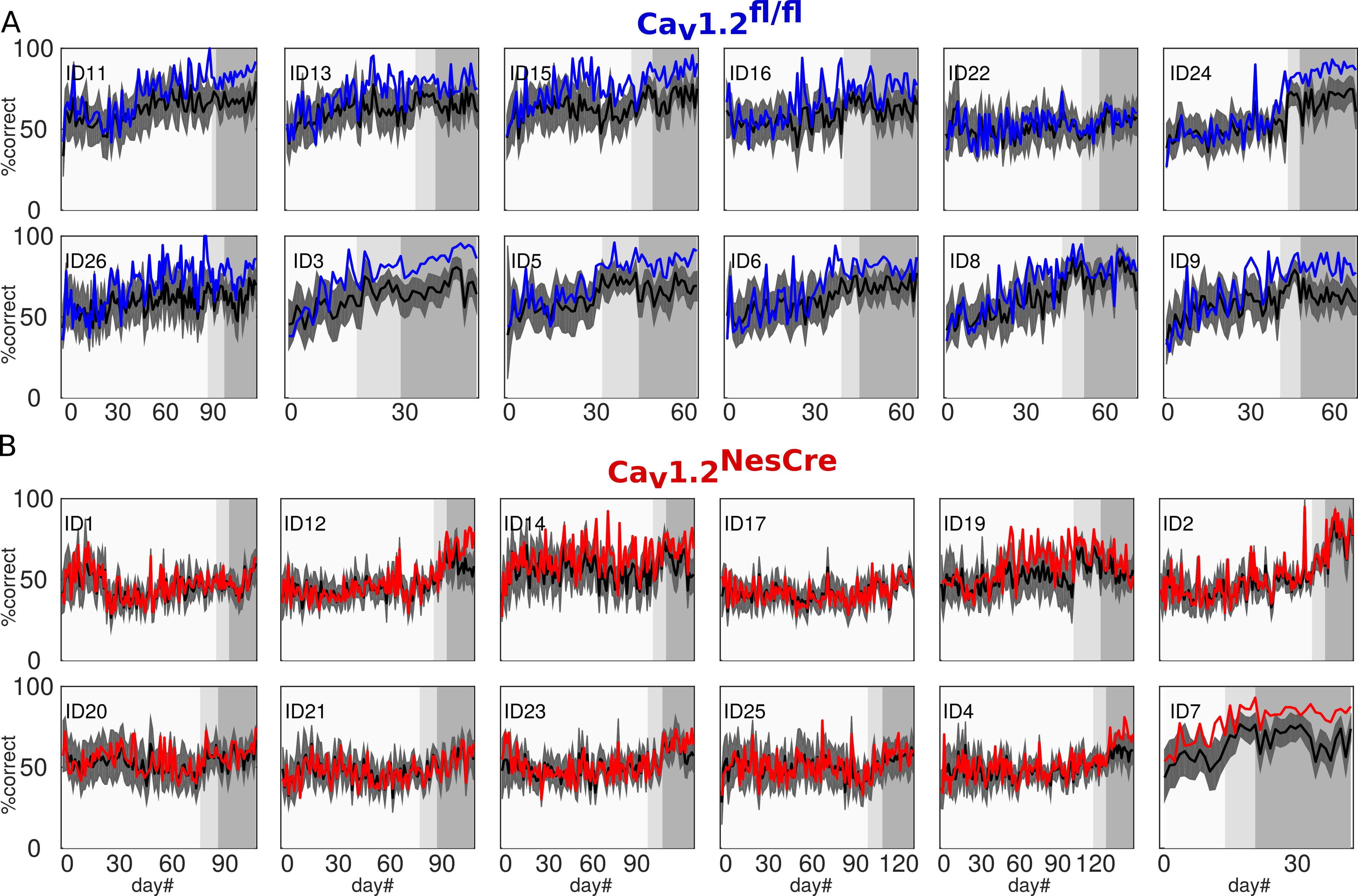

Supplement: S3 Fig — A) Actual performance (blue curves) and bootstrapped performance distributions (gray-shaded: 90% CI, black: mean) generated from the purely outcome-based response behavior that is most consistent with the animal’s actual behavior, i.e. with day-specific outcome-rule choice probabilities inferred from the animal’s actual distribution of outcome-rule-consistent responses. Curves and corresponding bootstrap distributions are shown for all Cav1.2fl/fl animals. B) Same for Cav1.2NesCre animals. Note that while most Cav1.2fl/fl mice escape the bootstrap distributions as the task progresses (later in phase I, or in phase II/III), most Cav1.2NesCre animals remain within the bootstrap 90%-confidence bounds. Data available at https://github.com/GKoppe/BehavioralData_Ana. (TIF) [file pbio.2000936.s003.tif]

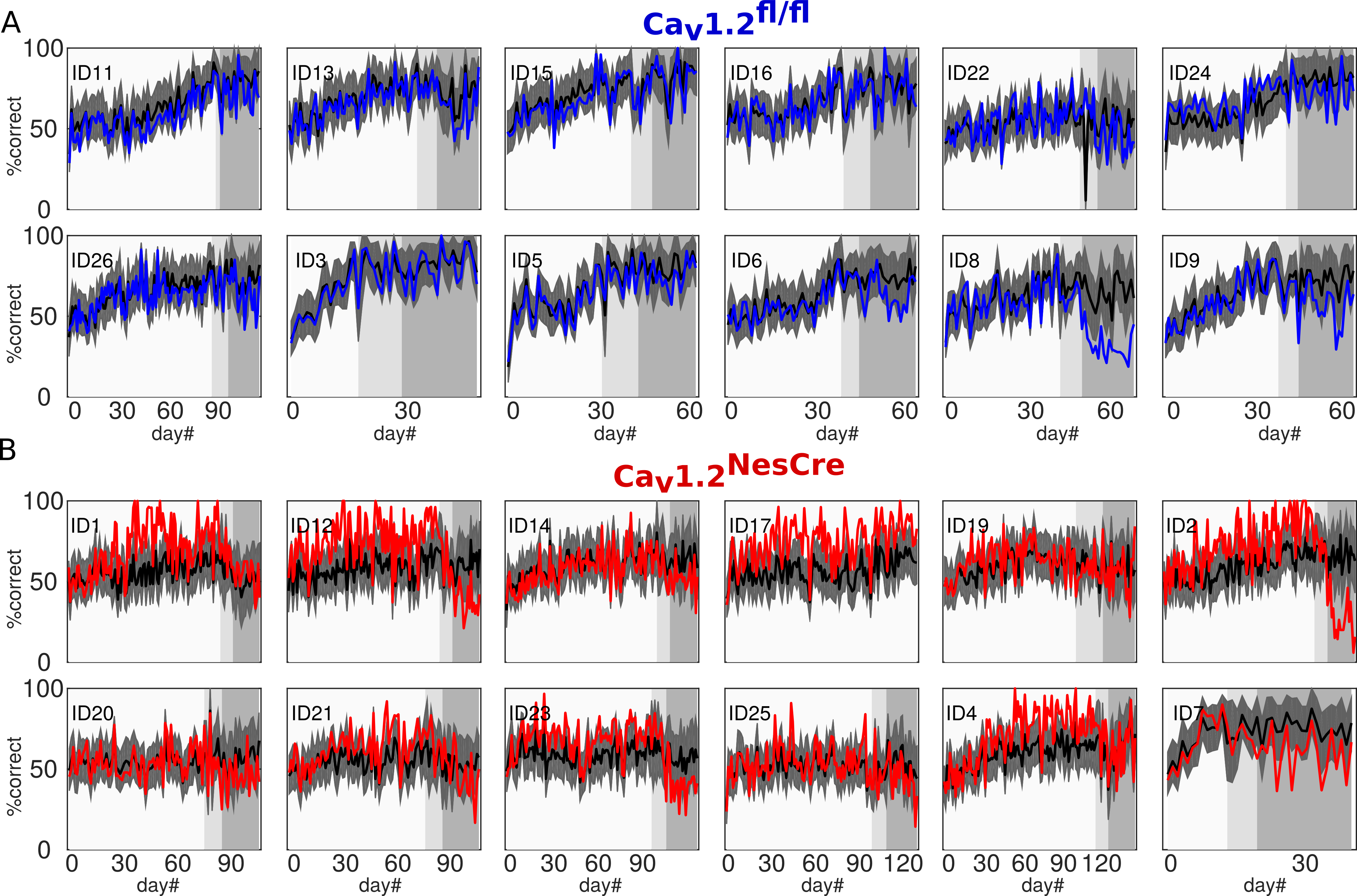

Supplement: S4 Fig — A) Actual performance (blue curves) and bootstrapped performance distributions (gray-shaded: 90% CI, black: mean) generated from the purely cue-based response behavior that is most consistent with the animal’s actual behavior, i.e. with day-specific cue-rule choice probabilities inferred from the animal’s actual distribution of cue-rule-consistent responses. Curves and corresponding bootstrap distributions are shown for all Cav1.2fl/fl animals. B) Cav1.2NesCre. Data available at https://github.com/GKoppe/BehavioralData_Ana. (TIF) [file pbio.2000936.s004.tif]

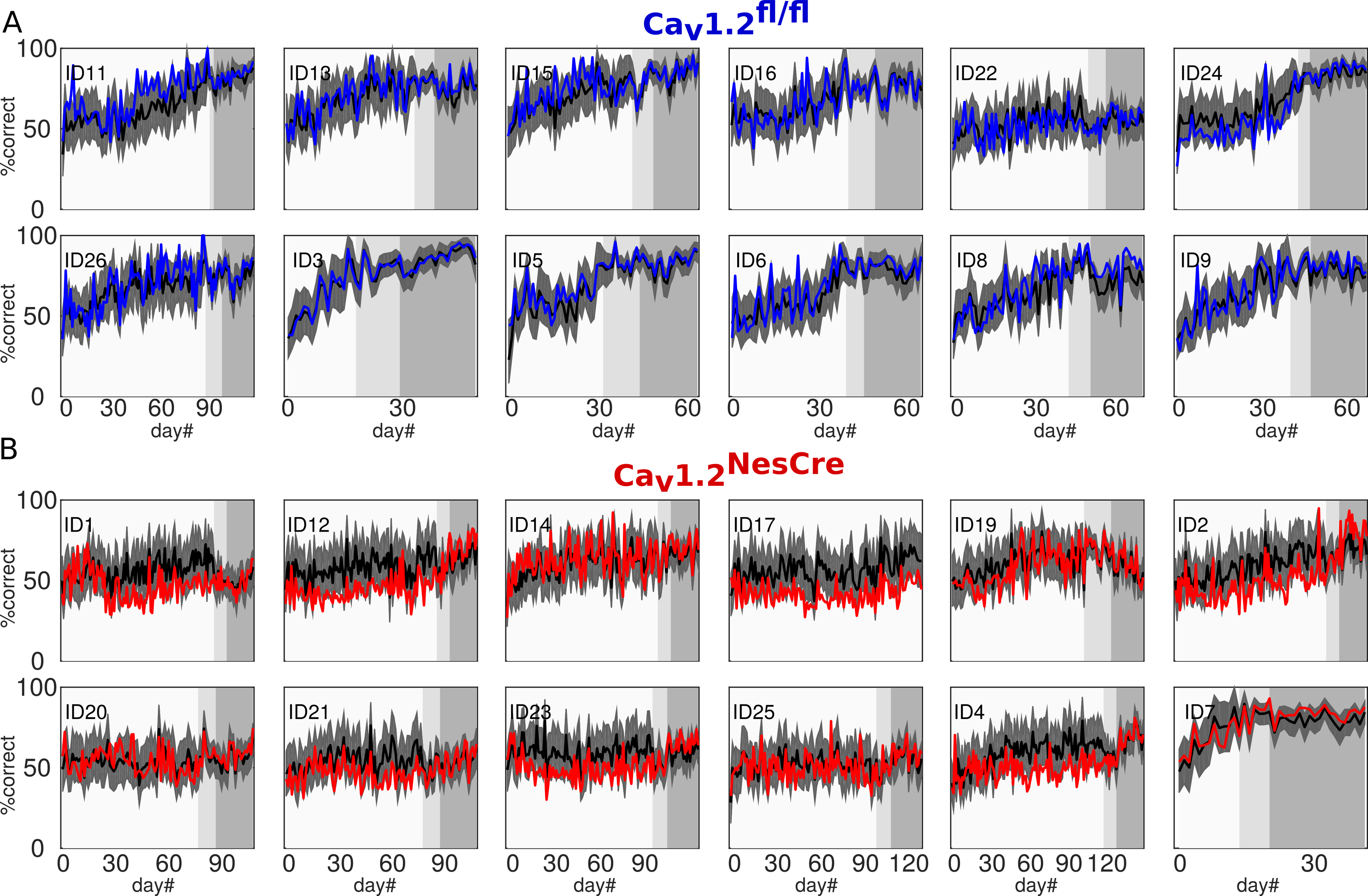

Supplement: S5 Fig — A) Actual performance (blue curves) and bootstrapped performance distributions (gray-shaded: 90% CI, black: mean) generated from the purely cue-based response behavior that is most consistent with the animal’s actual behavior, i.e. with day-specific cue-rule choice probabilities inferred from the animal’s actual distribution of cue-rule-consistent responses. Curves and corresponding bootstrap distributions are shown for all Cav1.2fl/fl animals. B) Same for Cav1.2NesCre. Data available at https://github.com/GKoppe/BehavioralData_Ana. (TIF) [file pbio.2000936.s005.tif]

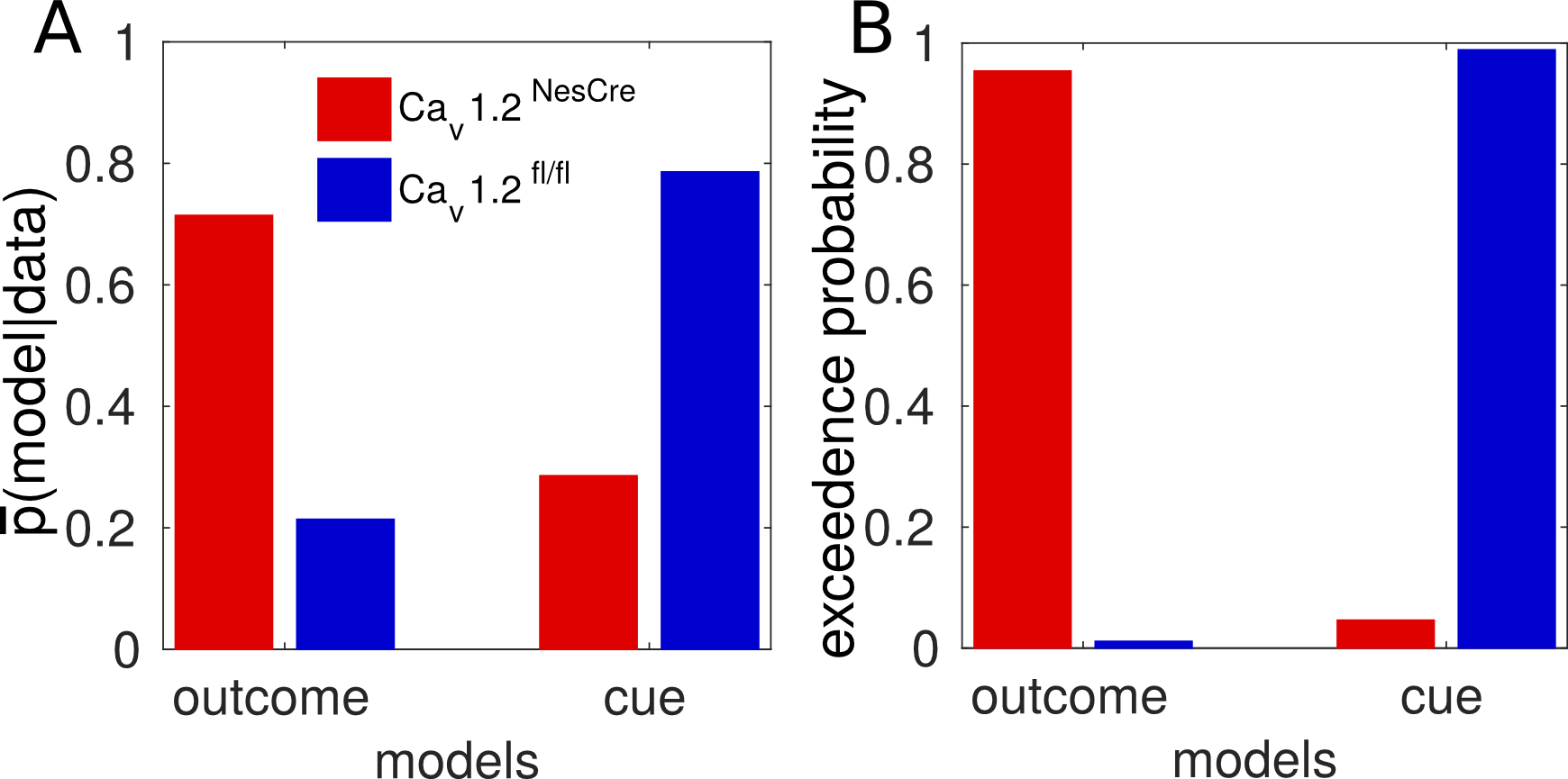

Supplement: S6 Fig — A) Expected posterior probabilities for cue and outcome model given Cav1.2NesCre and Cav1.2fl/fl data. B) Exceedance probabilities (i.e., the probabilities with which the posterior model probability of one model exceeds that of the other). (TIF) [file pbio.2000936.s006.tif]

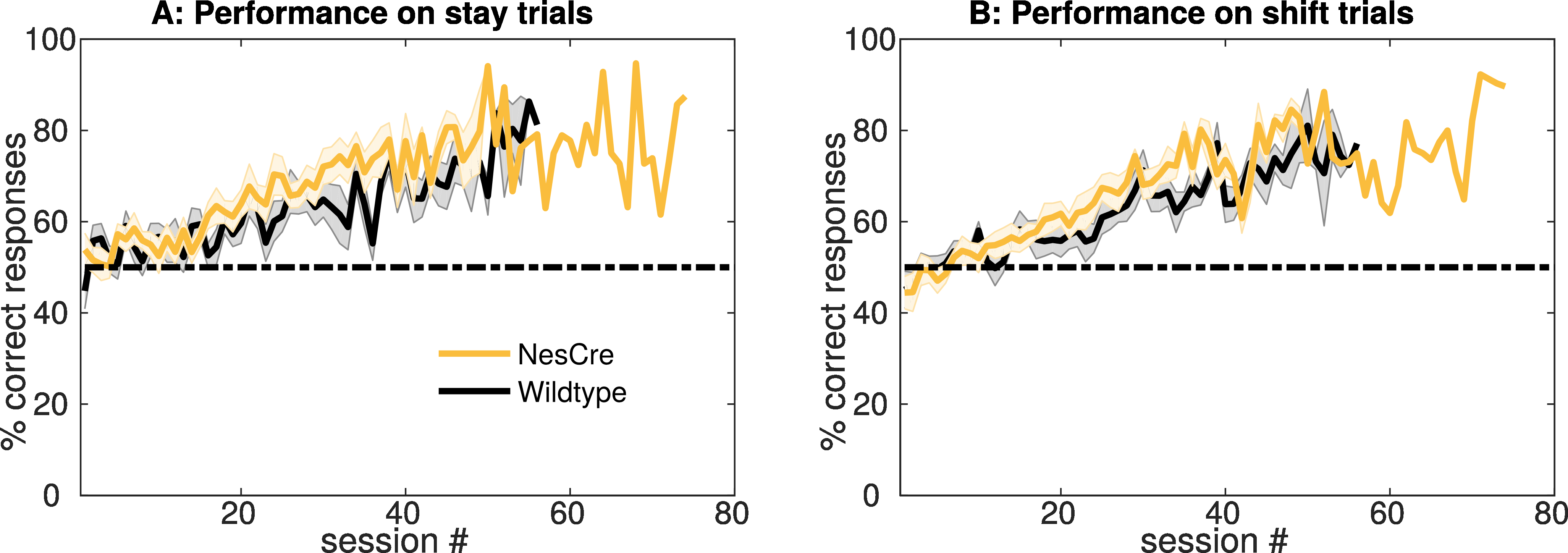

Supplement: S7 Fig — Mean and SEM of the percentage of correct responses by session for NesCre (yellow) and wild-type (black) mice for (A) stay trials, and (B) shift trials. In contrast to Cav1.2NesCre mice, both control groups exhibit a clear and significant improvement on stay trials during experimental phase I. On average, WT animals required 39.6 (+/- 11.5) and NesCre animals 40.3 (+/- 13.3) sessions to reach criterion. Data available at https://github.com/GKoppe/BehavioralData_Ana. (TIF) [file pbio.2000936.s007.tif]

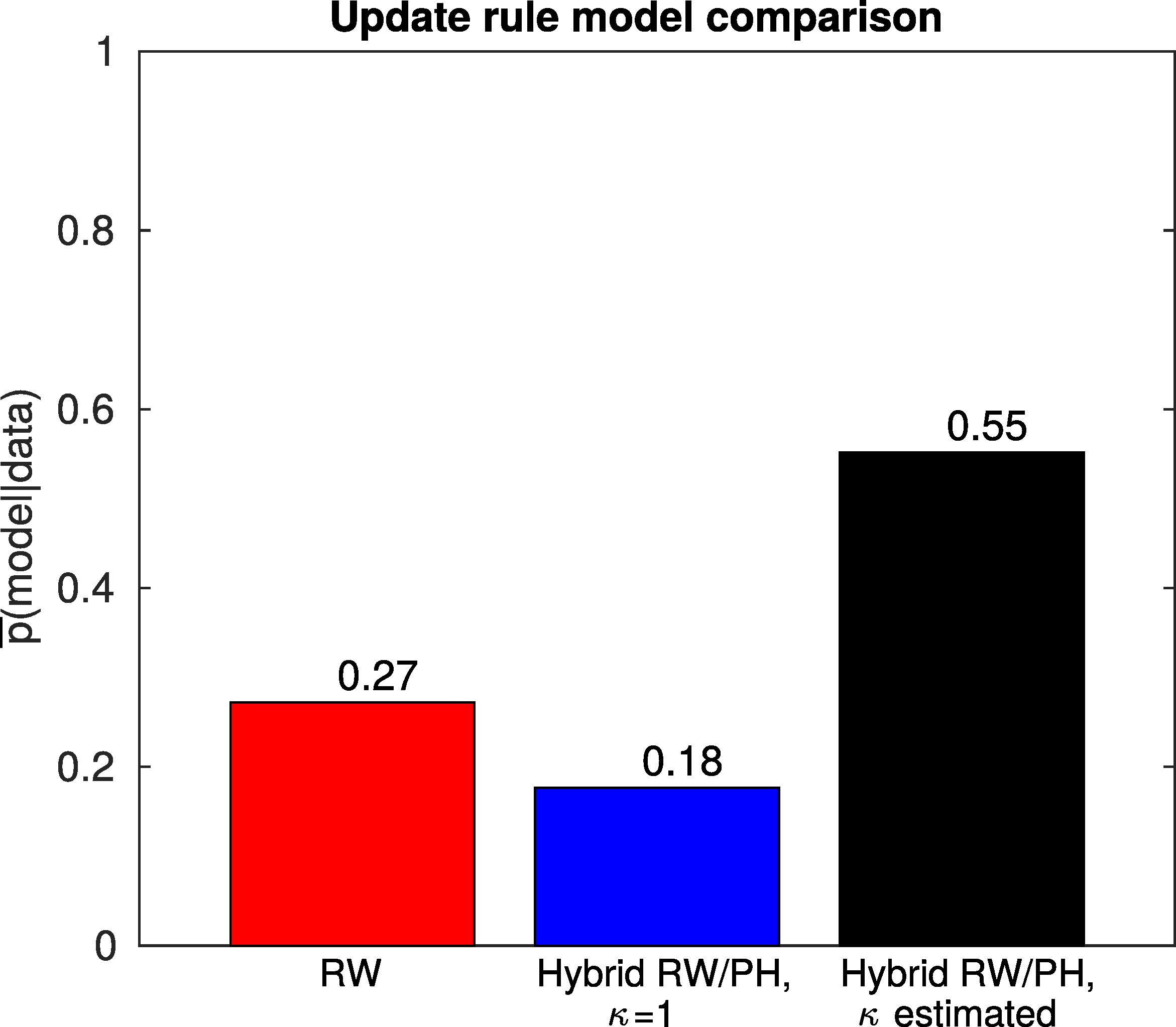

Supplement: S8 Fig — Expected posterior probabilities for a pure Rescorla-Wagner model, a hybrid model with a fixed global learning constant, and a hybrid model with the global learning rate parameter estimated from the data (see Materials and methods). Log model evidences that went into the model comparison were computed by averaging the BIC values across the cue and outcome models for each update rule. (TIF) [file pbio.2000936.s008.tif]
